# Integrating single-cobalt-site and electric field of boron nitride in dechlorination electrocatalysts by bioinspired design

**DOI:** 10.1038/s41467-020-20619-w

**Published:** 2021-01-12

**Authors:** Yuan Min, Xiao Zhou, Jie-Jie Chen, Wenxing Chen, Fangyao Zhou, Zhiyuan Wang, Jia Yang, Can Xiong, Ying Wang, Fengting Li, Han-Qing Yu, Yuen Wu

**Affiliations:** 1 CAS Key Laboratory of Urban Pollutant Conversion, Department of Environmental Science and Engineering, University of Science and Technology of China, 230026 Hefei, China; 2 Department of Applied Chemistry, Hefei National Laboratory for Physical Sciences at the Microscale, Collaborative Innovation Center of Chemistry for Energy Materials (iChEM), University of Science and Technology of China, 230026 Hefei, China; 3 College of Environmental Science and Engineering, Tongji University, 1239 Siping Road, 200092 Shanghai, China; 4 Beijing Key Laboratory of Construction Tailorable Advanced Functional Materials and Green Applications, School of Materials Science and Engineering, Beijing Institute of Technology, 100081 Beijing, China

**Keywords:** Pollution remediation, Heterogeneous catalysis, Electrocatalysis

## Abstract

The construction of enzyme-inspired artificial catalysts with enzyme-like active sites and microenvironment remains a great challenge. Herein, we report a single-atomic-site Co catalyst supported by carbon doped boron nitride (BCN) with locally polarized B–N bonds (Co SAs/BCN) to simulate the reductive dehalogenases. Density functional theory analysis suggests that the BCN supports, featured with ionic characteristics, provide additional electric field effect compared with graphitic carbon or N-doped carbon (CN), which could facilitate the adsorption of polarized organochlorides. Consistent with the theoretical results, the Co SAs/BCN catalyst delivers a high activity with nearly complete dechlorination (~98%) at a potential of −0.9 V versus Ag/AgCl for chloramphenicol (CAP), showing that the rate constant (*k*) contributed by unit mass of metal (*k*/ratio) is 4 and 19 times more active than those of the Co SAs/CN and state-of-the-art Pd/C catalyst, respectively. We show that Co single atoms coupled with BCN host exhibit high stability and selectivity in CAP dechlorination and suppress the competing hydrogen evolution reaction, endowing the Co SAs/BCN as a candidate for sustainable conversion of organic chloride.

## Abstract (web summary)

Bridging the biocatalytic repertoire and the effective environmental remediation remains a great challenge. Here, inspired by the dehalogenases, the authors designed a single atom Co catalyst on carbon doped boron nitride that exhibits high stability and selectivity in dechlorination.

## Introduction

Organic halides as the bulk commodity chemicals have been used broadly in medical industry, agriculture planting, and chemical engineering^1–3^. However, most of the halogenated compounds entering the environment are persistent with bioaccumulation potential in organisms and adverse effects on human health^4,5^. Among them, halogen-containing antibiotics, specially, chloramphenicol (CAP), a suspected carcinogen, was the first synthetic antibiotic to be applied at large scale since 1949^6,7^. The excessive use of antibiotics for a long time yields emerging antibiotic-resistant bacteria and antibiotic-resistant genes, becoming a growing public health problem^8,9^.

Dehalogenation generally enables to reduce the biological toxicity of halogenated compounds and also remove the antibiotic properties. However, traditional physicochemical techniques are often inadequate or expensive for breaking the strong carbon–chloride (C–Cl) bond in organic chloride, compared to bromide and iodide analogs^5,10^. The existing chemical reductive and oxidative dechlorination scenarios require long reaction times, high energy consumption, and/or costly or unstable reagents under particularly harsh reaction conditions and often lead to non-selective transformations, causing a secondary pollution^5,11^. Thus the development of strictly sustainable dehalogenation solutions to avoid jeopardizing the current natural resources has become an urgent challenge.

Biocatalytic dehalogenation on the basis of microorganisms typically operates under mild temperatures and pressures with fewer hazardous solvents in comparison of physicochemical transformations and plays a crucial role in achieving the sustainable development goals^12^. However, biocatalytic systems are time-consuming and incapable of degrading refractory and toxic pollutants completely^13–15^. To bridge nature’s catalytic repertoire and the demands of sustainable and effective environmental remediation, chemists have started to import function of microbial enzymes into chemical materials. In particular, the atomically dispersed single-metal-site materials might be an ideal platform to satisfy these demands due to the uniformity of isolated active site and the tunable coordination environment through the host structure^16,17^.

For dehalogenation, organohalide respiring bacteria with reductive dehalogenases (Rdh) composed of cobalt ionic center coordinated to tetrapyrrole-derived macrocycle could remove halogen through heterolytic cleavage from organic halides via the formation of halogen–cobalt intermediates^18,19^. The cobalt center and the binding pocket with optimal noncovalent interactions, generally including hydrogen bonding, electrostatic effects, *π*–*π* interaction, hydrophobic, and Van der Waals forces, are responsible for biological dehalogenation^20^. In addition, the electric field generated from the charged residues of the binding pocket also plays important roles in enzymatic dehalogenation. The local electric field around the active site could assist in activating the carbon–halogen bonds by changing the orientation and bond length of the organic halides^21–23^. Inspired by the intrinsic activity of Rdh, rationally tailoring the element compositions and molecular structure of the hosts for metal centers in single-metal-site materials is the key for the design of high-performance dechlorination catalysts^24^. Among the elements usually applied in the hosts, the electron-deficient B (2*s*^2^2*p*^1^) has fewer valence electrons than valence orbitals, readily sharing electrons donated from an electron-rich N (2*s*^2^*p*^3^). The resulting B–N bonds are partial ionic due to the large difference in electronegativity between B atom and N atom, presenting the local electric field^25^. Each functional B–N region with electric field could build up an overall electrostatic surface to interact with the C–Cl dipole, thus to capture the reactant correctly, like the function of the binding pocket in Rdh.

Inspired by the above analysis, based on electron localization function and partial density of states (PDOS) analysis, we report a design strategy to integrate enzyme-like single-atom sites with highly polarized support. Since electrocatalysis lies at the heart of green transformation of contaminants in the future technologies, single atomic Co confined in carbon-doped boron nitride (BCN) supports (Co SAs/BCN) as a robust and competent electrocatalyst for dechlorination was synthesized for proof-of-concept demonstration. Single Co sites behave as an enzyme-like active center. The electric fields endowed by the ionic B and N sites of Co SAs/BCN reinforce the interaction between Co SAs/BCN and organochlorides, thereby boosting dehalogenation kinetics. Computational studies reveal that Co SAs/BCN exhibit preferential and accelerated adsorption of chlorine than that of hydrogen. The single-atom Co catalyst delivers high electrocatalytic dechlorination performance for CAP, surpassing most of the reported heterogeneous catalysts in previously published studies (Supplementary Table 2).

## Results

### Electronic structure of Co SAs/BCN

**Fig. 1:**
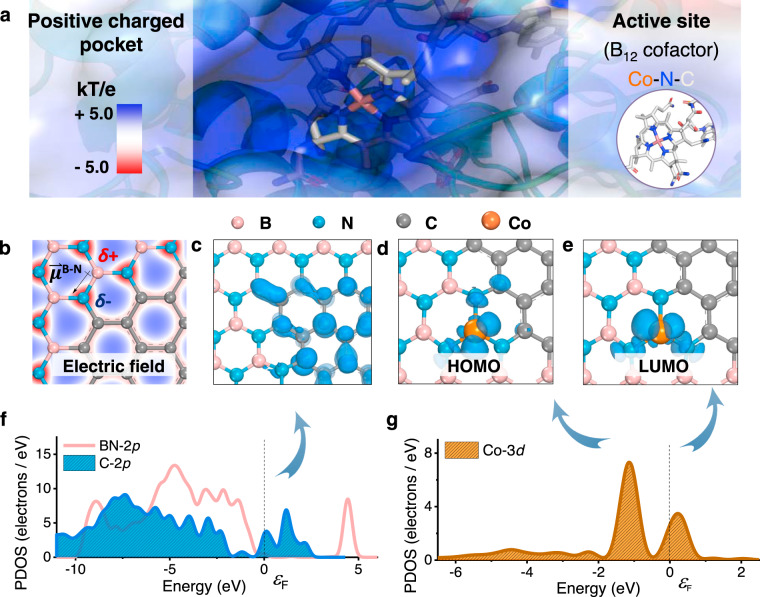
DFT calculations of Co SAs/BCN. **a** Electrostatic surface representation of the Rdh; the potential contour was scaled to +5.0 (blue) and −5.0 (red) *k*_B_*Te*^−1^ (where *k*_B_ is the Boltzmann constant, *T* is temperature, and *e* is the charge of an electron). **b** Electron density plots of BCN with the electric field around B–N bond; the isosurface contours reveal discontinuity in electron density between the N (negative charged) and B (positive charged) atoms. **c** Orbital density images of C atoms in BCN. **d** HOMO and **e** LUMO of isolated Co atom supported on BCN. **f** PDOS plot of BN-2*p* and C-2*p* orbitals. **g** PDOS plot of Co-3*d* orbitals; the peaks were imaged in **d**, **e**.

We first carried out density functional theory (DFT) calculations (detailed in Supplementary Methods) to analyze the feasibility of single-atom catalysts (SACs) to learn from the catalytic active sites and micro-environment of Rdh (Fig. 1a). Electron localization function analysis (Fig. 1b and Supplementary Fig. 1a) and Mulliken charge analysis (Supplementary Fig. 1b) show that the electron density is concentrated around N sites and lacks at B sites. This is in consistent with the results that BN is featured with ionic surface derived from its locally polarized B–N bonds^25^. The charge separation of BN could be similar to the micro-environment of the charged pocket in native enzymes, favoring the binding of organochlorides^26,27^. To evaluate the electrical conductivity, band structure of the pristine BN was calculated. The results show that BN exhibits as a semiconductor with a wide gap of 4.633 eV, impeding its electrochemical application. To improve the electrical conductance, the carbon atoms were doped into the BN substrate to form BCN. The band gap of BCN is significantly reduced to 0.084 eV by introducing new electronic states contributed by the carbon atoms (Fig. 1f)^28–30^. To evaluate the redox activity, charge analysis and orbital calculations were conducted. The atomic charge is a useful one to reflect the electron density map in solids for describing the distribution of electrons in chemical bonds. The Mulliken charge analysis is adopted to calculate the atomic charge through a linear combination of atomic orbital approach. The atomically dispersed Co sites anchored on BCN supports with net charge of 1.110 (Supplementary Fig. 1f), comparable with that of Co atom (1.03) in a simplified model of the active site in the reduced Rdh (Supplementary Fig. 2a)^20,31^. The highest occupied molecular orbital (Fig. 1d) of the isolated Co is near the Fermi level due to a positive shift of the energy levels (Fig. 1g and Supplementary Fig. 2b), which is beneficial for electron donating, suggesting a promising reducing ability of Co SAs/BCN.

Moreover, the binding of CAP on BCN is strictly stronger than that on nitrogen-doped carbon (CN) supports due to the additional electric field contributions (Supplementary Fig. 3). To demonstrate the interactions between Co SAs/BCN and CAP, PDOS analysis of Cl-2*p* orbitals and electronic structure calculations were conducted (Supplementary Fig. 4). The Cl-2*p* orbitals of free CAP molecule show one unoccupied energy band (P_1_) and one shallow occupied band (P_2_), corresponding to one anti-bonding orbital and one non-bonding orbital, respectively. When the CAP was adsorbed on Co SAs/BCN, a strong interaction was observed along with a newly occupied band (P_1′_), resulting from the electrons transferred from the Co-3*d* orbital to the Cl-2*p* orbital (Supplementary Fig. 4a). To relate the electron redistribution with the bond formation, electron density difference analysis was conducted. For the CAP and Co SAs/BCN complex, enriched electron density was found in the region between Cl and Co, giving rise to the Cl–Co bond (Supplementary Fig. 4c). This result further suggests that Co SAs/BCN should be very competitive for electrocatalytic dechlorination of CAP.

### Synthesis and structural characterization

**Fig. 2:**
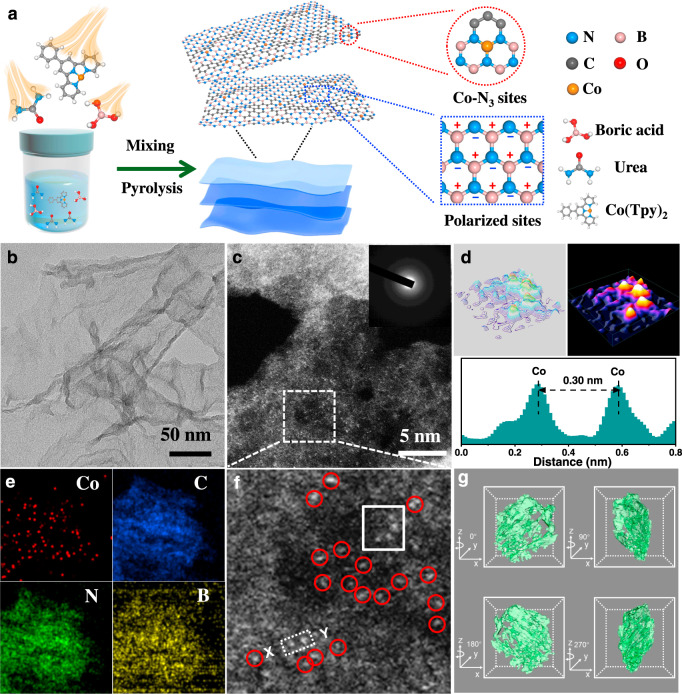
Preparation and characterization of Co SAs/BCN. **a** Schematic illustration of the formation of single-atom catalyst with atomically dispersed Co sites and polarized supports. **b** TEM image of Co SAs/BCN. **c**, **f** HAADF-STEM image of Co SAs/BCN (inset: SAED image). **d** 3D isolines and atom-overlapping Gaussian-function fitting mapping of the square from **f** and intensity profile along *X*–*Y* in **f**. **e** EELS element mapping. **g** Synchrotron X-ray three-dimensional computed tomographic images of Co SAs/BCN.

Inspired by the predicted superior structure, we try to synthesize the Co SAs/BCN catalysts with single cobalt sites and highly polarized BCN by a supermolecular controlled pyrolysis strategy (Fig. 2a). Briefly, a cobalt-based complex (Co(Tpy)_2_) was first obtained via a liquid phase coordination reaction approach. By using urea and boric acid as building units, supramolecular frameworks were fabricated via H-bonding attraction. The synthesis of Co SAs/BCN was achieved by simple thermal treatment of a mixture of cobalt complex and urea–boric acid architecture under an argon atmosphere. The initial loading of cobalt can be tuned by changing the ratio of metal complex added. Then single atomic Co species were trapped by the BCN materials, forming the isolated Co SAs/BCN catalysts. ICP-AES (inductively coupled plasma atomic emission spectroscopy) analysis revealed Co loadings of 1.4 wt.% for the resulted sample.

No characteristic crystal peaks of metal are observed in the powder X-ray diffraction (XRD) pattern of Co SAs/BCN sample (Supplementary Fig. 5), excluding the presence of any large crystalline particles of Co-containing species. The broad peaks at 25° and 43° are assigned to reflections of the (002) and (004) crystal face of graphitic carbon^32^, demonstrating its poor crystallinity. Likewise, no visible nanoparticles (NPs) are found in the scanning electron microscopic (SEM) (Supplementary Fig. 6a) and transmission electron microscopic (TEM) (Fig. 2b and Supplementary Fig. 6c) images. Co SAs/BCN possesses graphene-like nanosheet structure with large surface areas, which is verified by a specific Brunauer–Emmett–Teller (BET) surface area of 104.4 m^2^ g^−1^ (Supplementary Fig. 7). There are only two bands characteristic of carbon materials observed in the Raman spectra (G band at 1600 cm^−1^ and D band at 1350 cm^−1^), agreeing well with the aforementioned XRD results. The Co SAs/BCN and BCN samples have relatively high defects with *I*_D_/*I*_G_ = 1.16 and 1.12, respectively (Supplementary Fig. 8). This implies that the incorporation of B and N atoms would create abundant defect sites of carbon.

The aberration corrected high-angle annular dark-field scanning transmission electron microscopy (HAADF-STEM) with sub-Angstrom resolution was employed to identify the dispersion of Co species. As shown in Fig. 2c and Supplementary Fig. 9, high-density Co single atoms are uniformly dispersed in Co SAs/BCN sample, which are identified by isolated bright dots marked with red cycles (Fig. 2f). Additionally, low-magnified STEM image in dark fields indicates that there is no observable Co NPs in Co SAs/BCN (Supplementary Fig. 6d), in line with the ring-like selected-area electron diffraction pattern (Fig. 2c, inset). Three-dimensional (3D) isolines and atom-overlapping Gaussian-function fitting mapping (Fig. 2d) of the square from Fig. 2f further confirm that the Co sites are atomically dispersed. In addition, the intensity profile along *X*–*Y* in Fig. 2d displays that Co1 sites are separated by at least 0.3 nm. Electron energy-loss spectroscopy (EELS) was conducted to investigate the distribution of Co, B, N, and C (Fig. 2e), suggesting the homogeneous distribution of these elements over the entire architecture. Detailed 3D structure information of the Co SAs/BCN sample was visualized by using X-ray tomography (Supplementary Video 1). Cracks and wrinkles are identified in the sample (Fig. 2g), demonstrating noticeable anisotropy of the Co SAs/BCN topology. Similar to the preparation procedure of Co SAs/BCN, Co single atoms anchored on N-doped carbon were synthesized (Supplementary Fig. 10). Moreover, the atomic resolution HAADF-STEM images (Supplementary Fig. 11) confirm that the Co atoms in Co SAs/CN are also atomically dispersed.

**Fig. 3:**
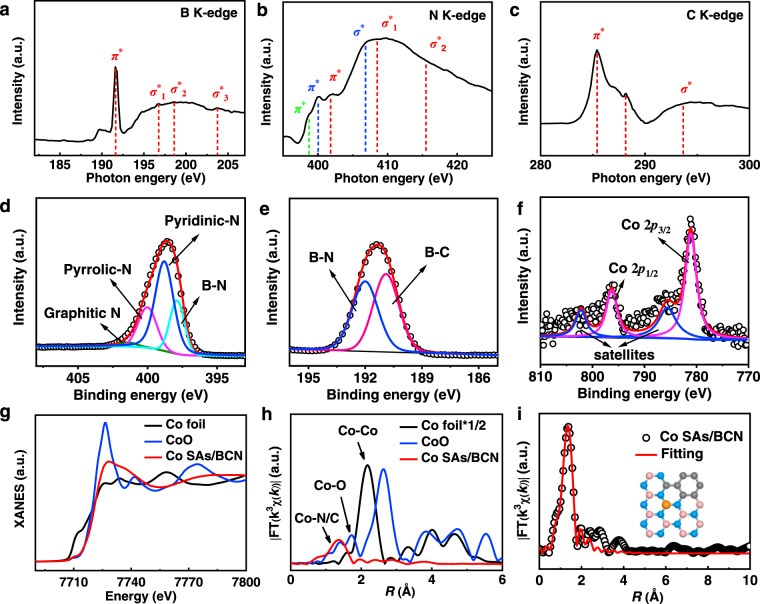
Chemical state and coordination information for Co SAs/BCN. **a** B *K*-edge, **b** N *K*-edge, and **c** C *K*-edge XANES spectra of Co SAs/BCN. **d**–**f** High-resolution XPS of N 1*s* (**d**), B 1*s* (**e**), and Co 2*p* (**f**) for Co SAs/BCN sample. **g** XANES spectra, **h** Fourier transform (FT) at the Co *K*-edge of Co SAs/BCN, CoO, and Co foil. **i** Corresponding EXAFS fitting curves of Co SAs/BCN at R space. The inset of **i** is the schematic model of Co SAs/BCN, Co (orange), N (blue), B (pink), and C (gray).

To gain a deep understanding on the electronic structures of Co SAs/BCN sample, we first resorted to the X-ray absorption near-edge structure (XANES) spectra to probe the types of nitrogen, boron, and carbon in the BCN structures. The B *K*-edge spectrum (Fig. 3a) shows the spectral fingerprints of B (*sp*^2^)–N (*sp*^2^) bonds with a sharp B 1*s* → π* at about 191.6 eV and three 1*s* → σ* resonances at 196.8, 198.6, and 203.7 eV^33^. These observations are strongly indicative of the formation of h-BN nanostructures. The presence of BN domains in Co SAs/BCN sample is further confirmed by N *K*-edge spectrum (Fig. 3b). These fingerprints of h-BN consist of π* resonance at 401.3 eV and two σ* features at 408.5 and 415.5 eV. Identical conclusions were obtained in literature findings that B and N atoms tend to form h-BN nanostructure due to their strong binding interactions, which is the most thermodynamically stable structure in BCN materials^34^. Meanwhile, the broadened N *K*-edge spectrum is associated with multiple forms of N species. Those peaks centered at 400 and 406.4 eV can be assigned to graphitic π* and σ* transitions. The peak at 398.7 eV suggests the presence of pyridinic or pyrrolic N^33,35^. These results strongly support the idea that Co SAs/BCN involves not only h-BN nanostructures but also a substantial amount of active N sites. For the C *K*-edge spectrum (Fig. 3c), these characteristic peaks at 285.4 and 293.6 eV are assignable to C=C π* (ring) excitations and C–C σ* (ring) transitions corresponding to graphitic carbon materials, respectively^36,37^. The peak at 288.1 eV suggests the formation of C–N–C or C–B σ* bond. It is reasonable to assume that the C dopant has been integrated into BN structure successfully.

For Co SAs/BCN (Supplementary Fig. 12), a substantial amount of C (45.2 at%) is doped into BN materials, calculated from X-ray photoelectron spectroscopy (XPS) analysis and confirmed by element analysis. The high-resolution N 1s spectrum can be principally deconvoluted into four types of N species (Fig. 3d), which correspond to N–B bonding in h-BN motifs (397.8 eV), pyridinic N (398.8 eV), pyrrolic N (400.0 eV), and graphitic N (401.4 eV)^38^. Similarly, the high-resolution XPS spectrum for B 1*s* (Fig. 3e) reveals that the dominant boron species are B–N and B–C species, respectively. Those XPS results further support that Co SAs/BCN materials consist of multiple types of B and N atoms. The Co 2*p* spectra (Fig. 3f) of Co SAs/BCN reveal that the binding energies of the Co 2*p*_3/2_ peaks at 781.1 eV with a satellite peak are characteristic of Co^2+^. This is consistent with the observation from previously reported results^39^. The survey spectrum and C high-resolution XPS spectrum of Co SAs/BCN were illustrated in Supplementary Fig. 13 to further analyze the chemical structure information.

X-ray absorption fine structure (XAFS) spectra were carried out to elucidate detailed structural information for the Co SACs. The XANES spectrum of Co SAs/BCN for describing the degree of oxidation (Fig. 3g) indicates that the valence of Co atom in Co SAs/BCN is inferred to be situated between Co^0^ and Co^2+^. This observation coincides with the aforementioned XPS results, implying the unique electronic structure of Co^δ+^ (0 < *δ* < 2). The oxidation state probed by the XPS and XANES measurements is helpful to identify whether an atom is being oxidized or reduced but does not represent the real charge of this atom. The extended XAFS (EXAFS) spectrum of R space for Co SAs/BCN displays a dominant Co–N coordination at 1.37 Å (Fig. 3h). Additionally, no characteristic peaks for Co–Co contribution at higher *R* value can be found, indicating that isolated cobalt atoms were distributed on the BCN supports. The quantitative coordination configuration of the Co atom in the Co SAs/BCN catalyst can be acquired by EXAFS fitting, as shown in Fig. 3i. The simulation reveals a main peak originating from Co–N/C first shell coordination. The coordination number of Co atoms is calculated to be about 3. The best-fit result of the EXAFS data is summarized in Supplementary Table 1.

### Electrocatalytic reductive dechlorination performance

The electrochemical reductive dechlorination activities of Co SAs/BCN, Co SAs/CN, and BCN were investigated by cyclic voltammogram (CV) and prolonged electrolysis in CAP solution. As revealed by the CV patterns in Supplementary Fig. 15, there are two reduction peaks of CAP on the blank glassy carbon electrode, consistent with the similar behavior in previous reports^40^. Co SAs/BCN displays high electrocatalytic activity for CAP reduction, achieving a reduction peak at −0.57 V vs. Ag/AgCl (all potentials are in reference to the Ag/AgCl). This value is more positive than those of Co SAs/CN (−0.64 V), Pd/C (−0.65 V, Supplementary Fig. 16), and BCN (−0.67 V).

**Fig. 4:**
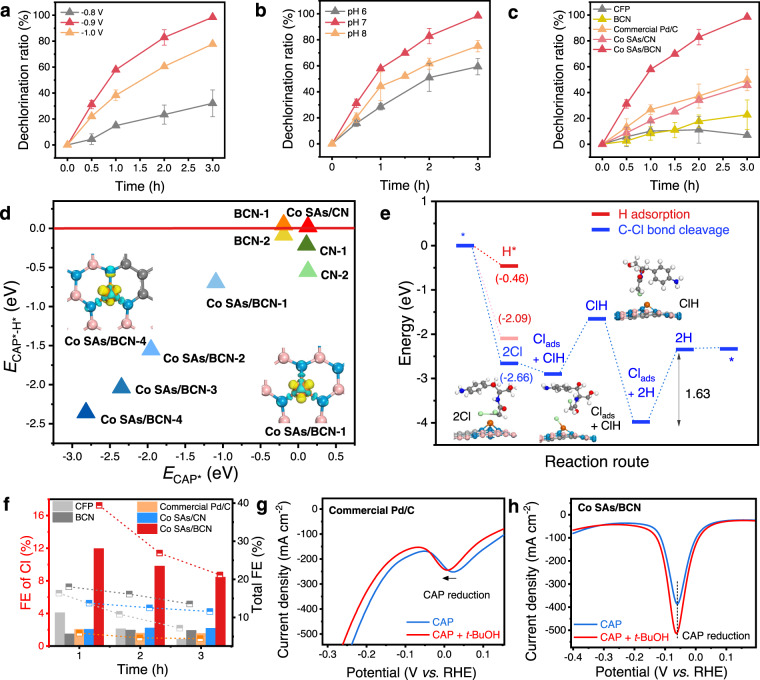
Catalytic mechanism of CAP electrocatalytic dechlorination. **a**, **b** Dechlorination ratio of CAP on various cathode potentials and at different pHs over the Co SAs/BCN, respectively. **c** Dechlorination ratio of CAP on various catalysts after 3 h electrolysis at −0.9 V. **d** Plot of the energy difference *E*_CAP*_ − *E*_H*_ against the *E*_CAP*_; detailed structures are given for BCN-1 and BCN-2 (Supplementary Fig. 22), Co SAs/BCN-1 to −4 (Supplementary Fig. 32), CN-1, CN-2, and Co SAs/CN (Supplementary Fig. 36). **e** Energy diagrams and intermediate states of CAP dechlorination on the Co SAs/BCN; the optimized structures are detailed in Supplementary Fig. 33. **f** Faradaic efficiency (FE) of CAP reduction. Left axis denotes the FE of CAP dechlorination (histogram). Right axis denotes the total FE of CAP reduction (dashed line), including the dechlorination reaction and the NO_2_ reduction of CAP. **g**, **h** Differential pulse voltammetry (DPV) curves of the commercial Pd/C and Co SAs/BCN, respectively, in solutions of 0.5 M H_2_SO_4_ with dose of CAP and *t*-BuOH.

To further analyze the reduction products, constant-potential electrolysis of CAP was carried out in a typical three-electrode H-type cell at different applied potentials. The conversion efficiency of CAP and released chloride ion was quantified by ultra-performance liquid chromatography (UPLC) and ion chromatography (IC), respectively. It is noted that the reduction efficiency achieved 89% in 1 h and 100% in 3 h with dechlorination efficiency of 58 and 98% at −0.9 V for Co SAs/BCN (Fig. 4a and Supplementary Fig. 17a). Meanwhile, for the Co SAs/CN catalyst without electric field effect (Fig. 4c), the dechlorination efficiency achieved 46% only in 3 h, demonstrating that the enhanced catalytic activity of Co SAs/BCN is derived from the electric field contribution. In addition, less chloride ions were detected after 3 h electrolysis on state-of-the-art Pd/C catalyst (50%). To rationally compare the catalytic activity of dechlorination catalysts, their faradaic efficiency (FE) of chloride ions were calculated. As depicted in Fig. 4f and Supplementary Figs. 30 and 31, the FE of chloride ions for Co SAs/BCN reached a maximum FE_Cl_ around 11.9% at −0.9 V, which surpassed Co SAs/CN and BCN during the dechlorination process. Impressively, the Co SAs/BCN can achieve aryl-NO_2_ reduction (FE_NO2_) and chorine removal concurrently at potentials of −0.9 V. Additionally, the surface area or electrochemically active area were measured to evaluate the electrochemical catalytic activity. The results show that the Co SAs/BCN exhibited the smallest electrochemical surface area (ECSA, Supplementary Fig. 35) and the smallest specific surface area (SSA, Supplementary Fig. 7) among the tested materials. Thus the Co SAs/BCN showed the highest electrochemical catalytic activity after normalizing to the ECSA or SSA. But the ideal way to compare the catalyst activity for SAC is to normalize the number of products with the number of catalytic active sites. However, until now, it is still technically difficult to accurately measure the number of catalyst active sites. Accordingly, rate constant (*k*) contributed by unit mass of metal (*k*/ratio) was used as a reasonable parameter for catalytic comparison (detailed in Supplementary Note 1). Thus the electrocatalytic dechlorination efficiency and CAP conversion rate constant (per metal/substrate mass ratio, *k*/ratio) of the various reductive dechlorination catalysts are compared in Supplementary Table 2. The value of Co SAs/BCN (605.8 h^−1^) is almost 19 times higher than that of Pd/C (32.0 h^−1^), indicating that Co SAs/BCN SACs outperformed most of current dechlorination reduction catalysts.

The electrocatalytic dechlorination activity of CAP apparently depends on the working potential. A more negative potential of −1.0 V slightly slowed down the dechlorination reaction due to the accelerated competing hydrogen evolution reaction (HER) reaction. However, at a more positive potential of −0.8 V, the dechlorination activity of Co SAs/BCN showed an obvious decline with only 47% CAP reduced. The effect of initial pH on CAP dechlorination was also examined. Co SAs/BCN in neutral solutions outperformed those under the acidic and basic conditions (Fig. 4b), which is beneficial for practical treatment of real wastewater. Subsequently, the influence of Co content in the catalyst was investigated in the subsequent tests (Supplementary Fig. 18a–b). As a result, a lower Co concentration (Co SAs/BCN-L) slowed down the reaction but still remained 95% CAP reduction efficiency and 76% dechlorination efficiency in 3 h. Co SAs/BCN-H showed the similar performance with Co SAs/BCN and achieved almost 100% chlorine removal by half hour early. Thus Co atom was supposed to be the active site as the electrocatalytic dechlorination efficiency for CAP shows a positive correlation with Co content.

The stability of the Co SAs/BCN during the electrochemical dechlorination process was evaluated. The results show that the Co ion would be released into the solution at a very low level. The electrochemical dechlorination performance could be maintained after five-time (15 h) electrolysis (Supplementary Figs. 19 and 20). The structure of the Co SAs/BCN was not changed after the test (Supplementary Fig. 21).

### Catalytic mechanism of CAP dechlorination

To gain further insights into the nature of the high activity of the Co SAs/BCN, the reaction free energies for CAP electrocatalytic dechlorination and the competing HER were investigated. As the Co site is believed to be the catalytic active center, Fig. 4e and Supplementary Figs. 32–34 show the calculated free energy diagrams and geometric structures in the dechlorination pathway on the Co site with the reasonable position (Supplementary Fig. 32). Generally, the reductive dechlorination of CAP involves concerted proton–electron transfer steps through heterolytic cleavage on surface metal sites, generating different intermediate adsorbates (the asterisk (*) denotes the surface site). At the same time, the HER was also considered since it might be the major competitive reaction (Supplementary Fig. 22). The initial step of HER is H adsorption (* + H^+^ + e^−^ → H*), which would block the catalyst surface and hinders CAP adsorption. However, in the same potential range, there was no significant current of HER and no signal of H* desorption on the CV with the Co SAs/BCN electrode (Supplementary Fig. 23b). This result indicates that the Co SAs/BCN was not beneficial to generate H*. Nevertheless, the Co SAs/BCN electrode showed a higher dechlorination capability and CAP conversion efficiency than the Pd/C electrode (Fig. 4c and Supplementary Fig. 25). Thus the dechlorination on the Co SAs/BCN electrode was independent on H*, which is different from the traditional hydrodechlorination mechanism on the Pd/C electrode. Moreover, the suppressed H* generation markedly inhibited the dechlorination performance on the Pd/C electrode (Fig. 4g) but had no effect on the dechlorination capability of the Co SAs/BCN (Fig. 4h, detailed description is given in Supplementary Note 2). At potential *U* = 0 V, Co SAs/BCN showed a selective adsorption of CAP* (−2.66 eV) over H* (−0.46 eV). For the subsequent conversion, the potential limiting step was suggested to be the removal of the adsorbed chloride ion after breaking C–Cl bond of CAP. Therefore, to drive the limiting step into thermodynamically feasible process, external potential was applied. At potential *U* = −1.63 V, each step of CAP dechlorination became spontaneous on Co SAs/BCN. The adsorption of CAP* was still preferred to H* (−2.09 eV), resulting in a dominant dechlorination reaction. On the other hand, the energy difference between CAP adsorption and H adsorption (i.e., *E*_CAP*_ − *E*_H*_) demonstrates as a reasonable descriptor of CAP reduction selectivity. More negative *E*_CAP*_ − *E*_H*_ corresponds to a higher selectivity toward CAP reduction. As shown in Fig. 4d, Co SAs/BCN exhibits more negative *E*_CAP*_ − *E*_H*_ in comparison with Co SAs/CN and BCN, consistent with the experimentally improved selectivity. Thus the proposed Co SAs/BCN indeed favors reductive dechlorination of CAP both from the DFT analysis and electrochemical measurements.

Simultaneously, the corresponding products of CAP by Co SAs/BCN were also determined by the high-performance LC (HPLC)/mass spectrometry (MS) (Supplementary Figs. 27 and 28). The main products were identified as nitroso products (*m*/*z* 305), hydroxylamine products (*m*/*z* 307), and aromatic amine products (*m*/*z* 281, *m*/*z* 247) after the steps of NO_2_ reduction and dechlorination (detailed in Supplementary Note 3).

In addition, compared to the Co SAs/CN, BCN, or CN support, the Co SAs/BCN had better dechlorination performance at the molecular level (Supplementary Fig. 24a, f). During the nitro group reduction (Supplementary Fig. 29a), the CAP adsorption makes the orientation of NO_2_ group toward Co active site with a binding energy of −1.826 eV, followed by the first hydrogenation step of NO_2_ group coupled with electron transfer (Supplementary Fig. 24b–e). The subsequent dissociation of OH forms the intermediate with NO group. The Co SAs/BCN presents a higher activation capability of the first N–O bond, which is the critical step in further reductive hydrogenation of nitro group. After the nitro group reduction, the dechlorination process is carried out via formatting a Co–Cl bond according to the heterolytic cleavage coupled with electron transfer (Supplementary Fig. 29b). The results show that the free energy profiles of the first cleavage of C–Cl bond on the Co SAs/BCN become downhill with the most released energy (Supplementary Fig. 24f–j), which could be used to drive the subsequent elementary reactions, indicating that dechlorination is more favorable on the Co SAs/BCN. Thus the electrocatalytic dechlorination mechanism of the Co SAs/BCN is different from the hydrodechlorination mechanism on the Pd/C but obeys the process of heterolytic cleavage coupled electron transfer.

## Discussion

We have demonstrated that enzyme-like active sites and electric field could be incorporated into an artificial catalyst by anchoring Co single site on BCN supports. The Co single site was identified as a highly active enzyme-like site for electrocatalytic dechlorination. The locally polarized B–N bonds play a key role in exerting electric fields onto C–Cl, contributing organochlorides adsorption on Co SAs/BCN. The as-fabricated Co SAs/BCN catalyst displays a good activity in CAP reductive dechlorination, and dechlorination efficiency can reach up to around 98%, which is a significant enhancement by a factor of 2 in comparison to the SAC of Co SAs/CN without electric field effect. Furthermore, the well-defined active site of atomically dispersed Co catalyst is further understood by control experiments and detailed DFT calculations. Our work suggests that SACs might open up opportunities for the design of high-performance enzyme-like catalysts.

## Methods

### Chemicals

All the chemicals were of analytical grade and used without further purification. p-Anisaldehyde, 2-acetylpyridine, potassium hydroxide, ammonia, ethanol, boric acid, and urea were purchased from Sinopharm Chemical Reagent Beijing Co., Ltd. (China). Cobalt (II) acetate, polyether F127, chloramphenicol, and commercial Pd/C (5 wt.%) were obtained from Aladdin Company. Chloramphenicol was obtained from Sigma-Aldrich. Deionized (DI) water (*R* = 18.25 MΩ) was used in all the experiments.

### Synthesis of cobalt complex

Briefly, a 20 ml vial was charged with 0.352 g (1 mmol) of substituted terpyridine in 10 ml of tetrahydrofuran. In all, 0.177 g (1 mmol) of anhydrous Co(OAc)_2_ was added to the yellow solution stirring for 24 h at room temperature. The resultant orange precipitate was centrifuged and then washed for several times with pentane. Finally, the obtained Cobalt complex was dried in a vacuum oven for further use.

### Synthesis of Co SAs/BCN

For single-atom Co supported on BCN catalyst preparation, a certain mass ratio of boric acid and urea (0.1 g of H_3_BO_3_ mixed with 3 g of urea) was dissolved in a mixture of DI water (5 ml) and ethanol (5 ml), followed by addition of 10 mg prepared Cobalt complex and 0.2 g F127. Then the homogeneous solution was heated under 80 °C for recrystallization. An orange crystalline powder product subsequently formed with the evaporation of solvents. Then the obtained orange powder was moved in a crucible with a cap and transformed to a tube furnace. Then the precursors were heated with a ramping rate of 5 °C min^−1^ to 800 °C and kept for 120 min under protection of Ar. After that, the tube furnace was naturally cooled to room temperature. Samples with different cobalt contents were also synthesized under otherwise identical conditions. They are denoted as Co SAs/BCN, Co SAs/BCN-L, and Co SAs/BCN-H, respectively.

### Synthesis of Co SAs/CN

For single-atom Co supported on nitrogen-doped carbon catalyst preparation, 0.1 g of glucose and 3 g of urea was dissolved in a mixture of DI water (5 ml) and ethanol (5 ml), followed by addition of 10 mg prepared Cobalt complex and 0.2 g F127. Then the homogeneous solution was heated under 80 °C for recrystallization. An orange crystalline powder product subsequently formed with the evaporation of solvents. Then the obtained powder was moved in a crucible with a cap and transformed to a tube furnace. Then the precursors were heated with a ramping rate of 5 °C min^−1^ to 800 °C and kept for 120 min under protection of Ar. After that, the tube furnace was naturally cooled to room temperature.

### Synthesis of BCN

For the BCN catalyst preparation, a certain mass ratio of boric acid and urea (0.1 g of H_3_BO_3_ mixed with 3 g of urea) was dissolved in a mixture of DI water (5 ml) and ethanol (5 ml), followed by addition of 0.2 g F127. Then the homogeneous solution was heated under 80 °C for recrystallization. A white crystalline powder product subsequently formed with the evaporation of solvents. Then the obtained orange powder was moved in a crucible with a cap and transformed to a tube furnace. Then the precursors were heated with a ramping rate of 5 °C min^−1^ to 800 °C and kept for 120 min under protection of Ar. After that, the tube furnace was naturally cooled to room temperature.

### Characterizations

Powder XRD patterns of samples were recorded on a Rigaku Miniflex-600 operating at 40 KV voltage and 15 mA current with Cu Kα radiation (*λ* = 0.15406 nm). The SEM images were taken using a field-emission SEM (JSM-6701F, JEOL) operated at an accelerating voltage of 5 kV. The morphologies of samples were examined by TEM, using a Hitachi-7700 microscope with an accelerating voltage of 100 kV. The high-resolution TEM, HAADF-STEM, and EELS mapping were carried out by JEOL JEM-ARM200F TEM/STEM with a spherical aberration corrector working at 200 kV. The XPS was carried out on a Perkin-Elmer RBD upgraded PHI-5000C ESCA system. Raman scattering spectra were performed with a Renishaw System 2000 spectrometer using the 514.5 nm line of Ar^+^ for excitation. Elemental analysis of Co in the solid samples was detected by an Optima 7300 DV ICP-AES. The obtained adsorption–desorption isotherms were performed on a Micromeritics Tristar II 3020 M to evaluate the BET-specific surface area.

#### XAFS measurement and data analysis

The X-ray absorption fine structure data (Co *K*-edge) were collected at the beamline 1W1B station of the Beijing Synchrotron Radiation Facility, China. The Co *K*-edge XANES data were recorded in a fluorescence mode. The storage ring was working at the energy of 2.5 GeV with an average electron current of 250 mA. The hard X-ray was monochromatized with Si (111) double crystals. The acquired EXAFS data were extracted and processed according to the standard procedures using the ATHENA module implemented in the IFEFFIT software packages. The k3-weighted EXAFS spectra were obtained by subtracting the post-edge background from the overall absorption and then normalizing with respect to the edge-jump step. Subsequently, k3-weighted *χ*(*k*) data in the *k*-space ranging from 2.5 to 11.2 Å^−1^ were Fourier transformed to real (*R*) space using a Hanning window (dK = 1.0 Å^−1^) to separate the EXAFS contributions from different coordination shells. Soft X-ray absorption spectra (C *K*-edge, N *K*-edge, and B *K*-edge) were carried out at the Catalysis and Surface Science Endstation at the BL11U beamline in the National Synchrotron Radiation Laboratory (NSRL) in Hefei, China. The soft X-ray computed tomography was conducted on the beamline BL07W of NSRL. For the tomographic reconstruction, the projections were reconstructed by using the total variation-based simultaneous algebraic reconstruction technique (SART-TV). Then the reconstructed images were imported into Amira (FEI Visualization Sciences Group, MA) for segmentation and 3D visualization.

### Electrochemical measurements

All electrochemical measurements were conducted using a CHI 760D Potentiostat in three-electrode configuration. An H-type electrochemical cell was employed with cathode cell (100 ml) and anode cell (100 ml) separated by the proton exchange membrane (Nafion-117, Du Pont). A platinum wire of 0.5 mm diameter was used as the counter electrode. All potentials were measured against a saturated Ag/AgCl reference electrode. A glassy carbon electrode (GCE) of 3 mm diameter was used as the working electrode for CV measurements. Before catalyst modification, the GCE was well polished with 0.05 μm alumina slurry. The carbon fiber paper (CFP, thickness 0.28 mm, TGP-H-090, Toray) was used as the working electrode for constant-potential electrolysis. Catalyst ink was prepared by dispersing 2 mg of catalyst material in a mixture of 50 μl of 5 wt.% Nafion solution, 200 μl of DI water, and 300 μl of ethanol with the assistance of sonication. An aliquot of 6 μl of the catalyst ink was applied onto the GCE and allowed to dry in air, giving a catalyst loading of 0.34 mg cm^−2^. By drop-drying of 200 μl of the catalyst ink on CFP (1.0 cm × 2.5 cm), the loading was 0.32 mg cm^−2^. The supporting electrolyte utilized for all experiments were prepared by using 0.067 M Na_2_HPO_4_ and KH_2_PO_4_ solutions. CV measurements were performed in an N_2_-saturated electrolyte to avoid interference from dissolved O_2_. During the controlled potential electrolysis, the electrolyte was agitated with a stirring bar at a stirring rate of about 350 rpm.

Total FE of CAP reduction was calculated by FE = FE_Cl_ + FE_NO2_, where FE_Cl_ is the partial FE of CAP dechlorination (C–Cl → C–H) and FE_NO2_ is the partial FE of NO_2_ reduction (NO_2_ → NH_2_). Partial FE of dechlorination is calculated by FE_Cl_ (*t*) = ratio (*t*)·4*cVF*/*Q*, where *c* is the concentration of chloride ions by 100% dechlorination, 4 denotes the four electrons required for dechlorination (CAP-2Cl + 2H^+^ + 4e^−^ → CAP-2H + 2Cl^−^), and ratio (*t*) is the dechlorination ratio of CAP at reaction time *t*. Analogously, FE_NO2_ (*t*) = ratio(*t*)·6*cVF*/*Q*, where *c* is the concentration of NO_2_ by 100% conversion and 6 denotes the six electrons required for reduction of NO_2_ group (NO_2_ + 6H^+^ + 6e^−^ → NH_2_ + 2H_2_O).

### Analytical methods

The extent of CAP reduction was quantified by determining the concentrations of CAP with ultra-high-pressure LC (Agilent 1290 Infinity, USA) equipped with a C18 column (2.1 mm × 50 mm, 1.8 μm). The mobile phase was acetonitrile and 0.1 wt.% HCOOH in a 1:4 ratio by volume with a flow rate of 3.0 ml min^−1^. The concentration of chloride ions released was measured by IC (ICS-1000, Dionex, Sunnyvale, CA). The mobile phase was 9.0 mM Na_2_CO_3_ with a flow rate of 1.0 ml min^−1^. The chloride ions in the background electrolyte was determined and abstracted to give a net measurement of the [Cl^−^] in the sample.

The products in solution were analyzed using LC-MS (XEVO G2-XS, QTOF, Waters Inc., USA), which was equipped with a UPLC system and an electron spray ionization (ESI) source. A Waters C18 column (4.6 mm × 50 mm, 1.7 µm particle size) was used for UPLC separation. The mixture of acetonitrile and H_2_O (containing 0.1% formic acid) was used as the mobile phase, and the flow rate was set at 0.4 ml min^−1^. An isocratic elution method was used in the separation procedure, which was kept at 20:80 (acetonitrile: H_2_O) for 5 min. An ESI source in the positive ionization mode was used for MS analysis. The mass calibration range was between 50 and 1200 Da, and the resolution was kept >30,000.

## Supplementary information

Supplementary Information

Description of Additional Supplementary Files

Supplementary Movie 1

## Data Availability

The data supporting the findings of this study are available within the article and its Supplementary Information files. All other relevant source data are available from the corresponding authors upon reasonable request.
