# Supplementary material for: Integrating single-cobalt-site and electric field of boron nitride in dechlorination electrocatalysts by bioinspired design
Source: Nat Commun. 2021 Jan 12;12:303. doi: 10.1038/s41467-020-20619-w (PMC7803959; doi:10.1038/s41467-020-20619-w)
Supplement: Supplementary file 1 — Supplementary Information [file 41467_2020_20619_MOESM1_ESM.pdf]

Supplementary Information for

Integrating single-cobalt-site and electric field of boron nitride in dechlorination electrocatalysts by bioinspired design

Min *et al.*

Supplementary Figures and Tables (2-40)

Supplementary Notes (41-44)

Supplementary Methods (45-46)

Supplementary References (47-48)

Supporting Figures and Tables

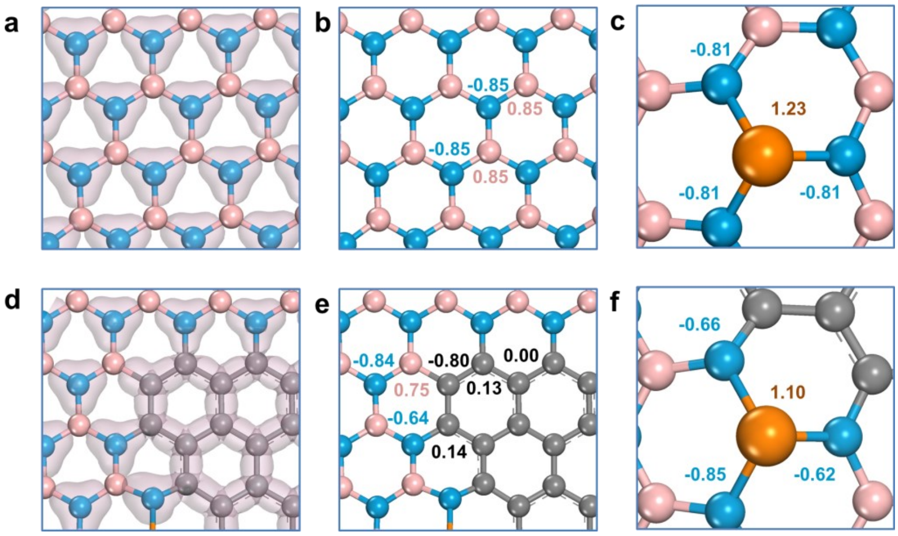

Supplementary Figure 1. Electron distribution on BN, BCN, Co SAs/BN, and Co SAs/BCN. a, d, Electron density plots of BN and BCN, respectively. The isosurface level is ±0.005 e $Bohr^{-3}$. **b, c, e, f,** Mulliken charges of BN, the supported Co atom, BCN, and the supported Co atom for describing the distribution of electrons in chemical bonds, respectively.

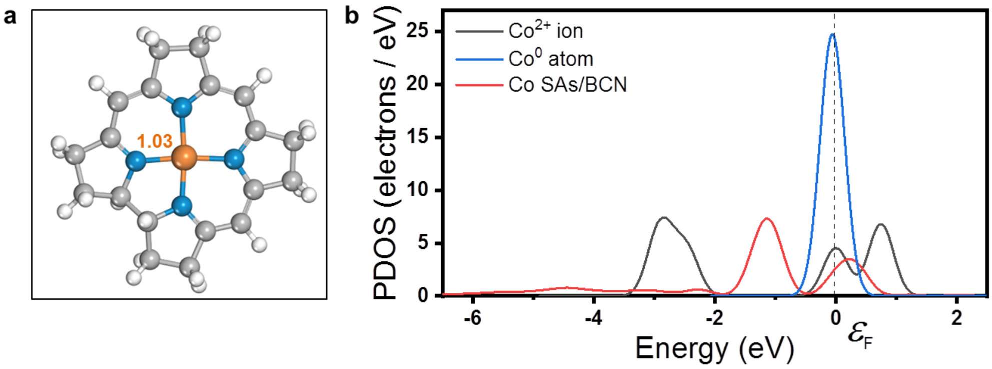

Supplementary Figure 2. Atomic charge of Co atom in the active site of the reduced Rdh and density of states of Co in different coordination environments. **a,** Atomic charge of Co atom in a corrin ring, a simplified model for the active site of the reduced Rdh, from Mulliken charge analysis. **b,** Partial density of states (PDOS) plots of Co-3*d* orbitals of Co^{2+} ion, Co atom, and the Co anchored in BCN, respectively. The valence of Co in Co SAs/BCN is located between Co^0 and Co^{2+}, suggesting a promising reducing ability.

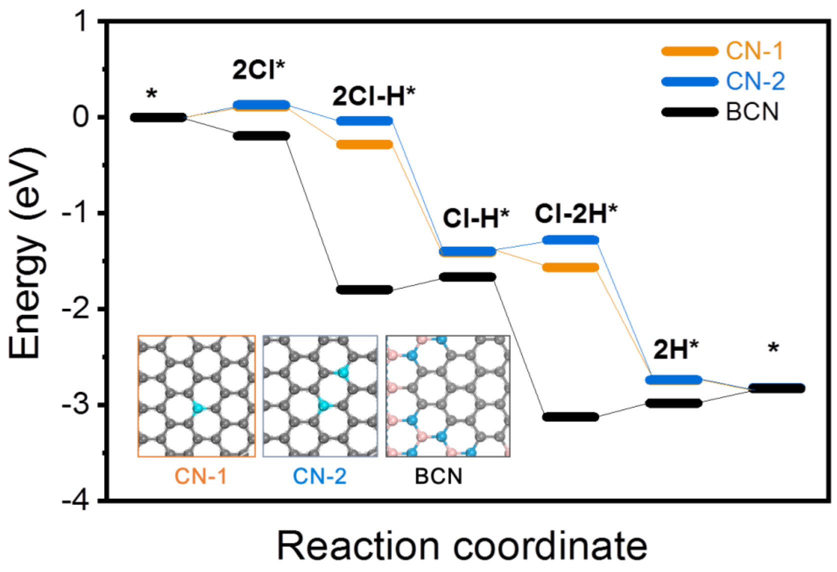

Supplementary Figure 3. Energy diagrams of CAP dechlorination on CN and BCN.

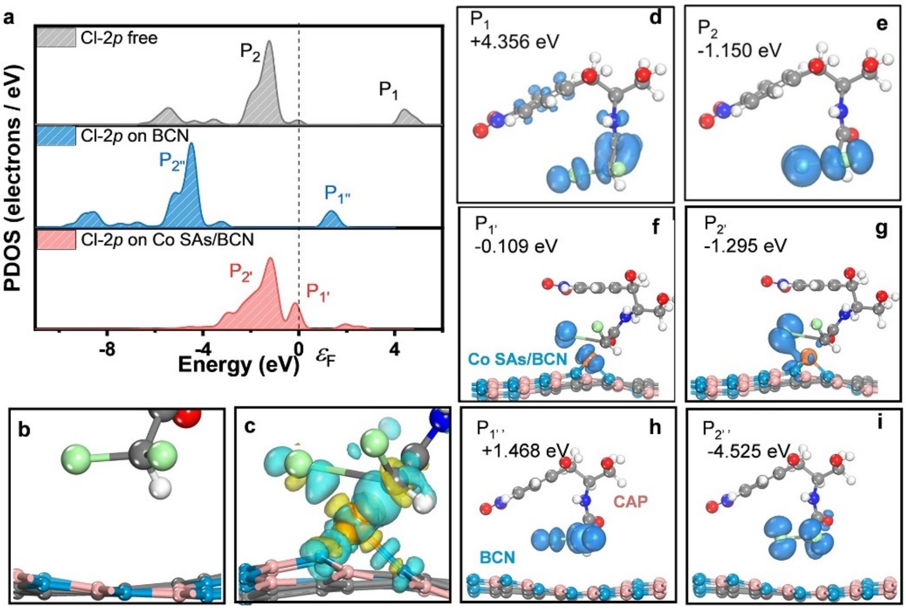

Supplementary Figure 4. PDOS, electron density difference plots and orbital density images. a, PDOS plot of Cl-2*p* orbitals of free CAP molecule (gray), CAP adsorbed on BCN (blue) and CAP adsorbed on Co SAs/BCN (red). Electron density difference plots of the interface between (**b**) CAP and BCN, and (**c**) CAP and Co SAs/BCN, respectively. The isosurface level is ±0.1 electrons/Å^3. **d, e,** Orbital density images of the free CAP molecule, peak P_1 (energy level: +4.356 eV), peak P_2 (energy level: -1.150 eV), respectively. **f, g,** Orbital density images of the peak $P_{1'}$ (energy level: -0.109 eV) and peak $P_{2'}$ (energy level: -1.295 eV) on Co SAs/BCN. **h, i,** Orbital density images of the peak $P_{1''}$ (energy level: +1.468 eV) and peak $P_{2''}$ (energy level: -4.525 eV) on BCN.

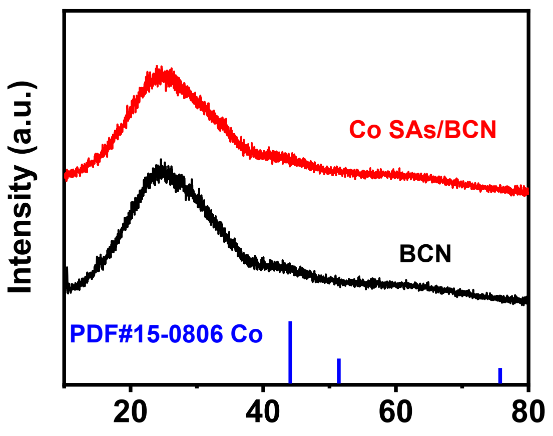

Supplementary Figure 5. XRD patterns of BCN and Co SAs/BCN.

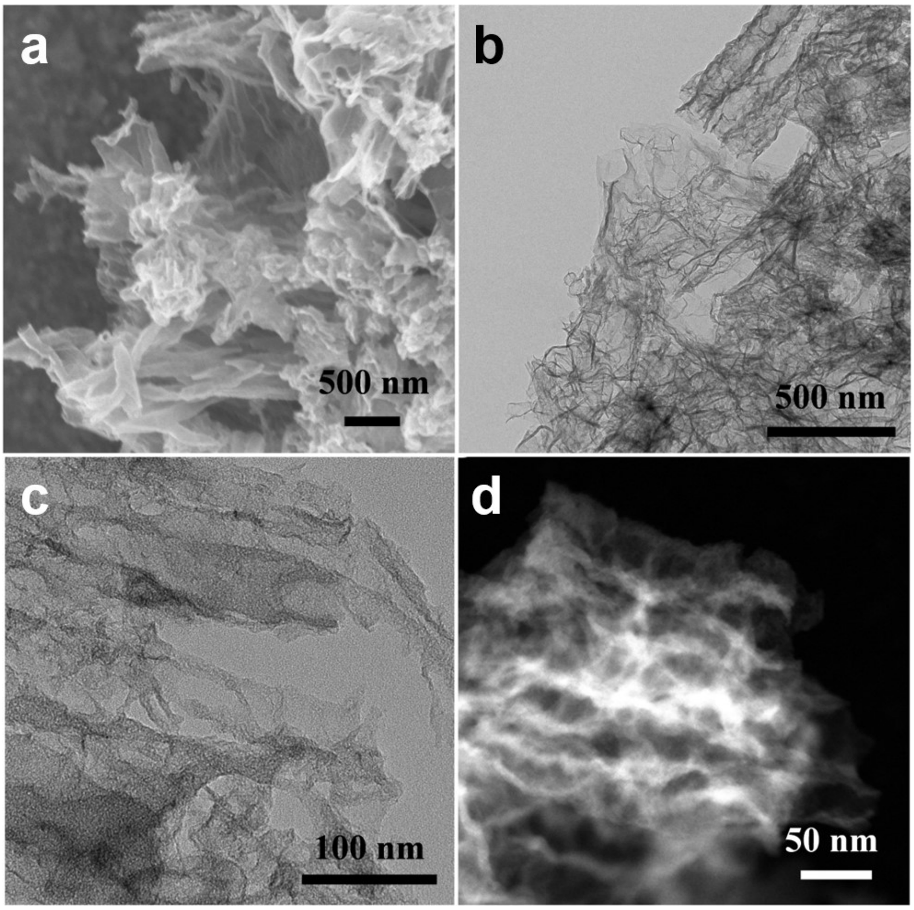

Supplementary Figure 6. Morphology characterizations of Co SAs/BCN. a, Representative SEM, **b, c,** TEM, and **d,** STEM images of Co SAs/BCN.

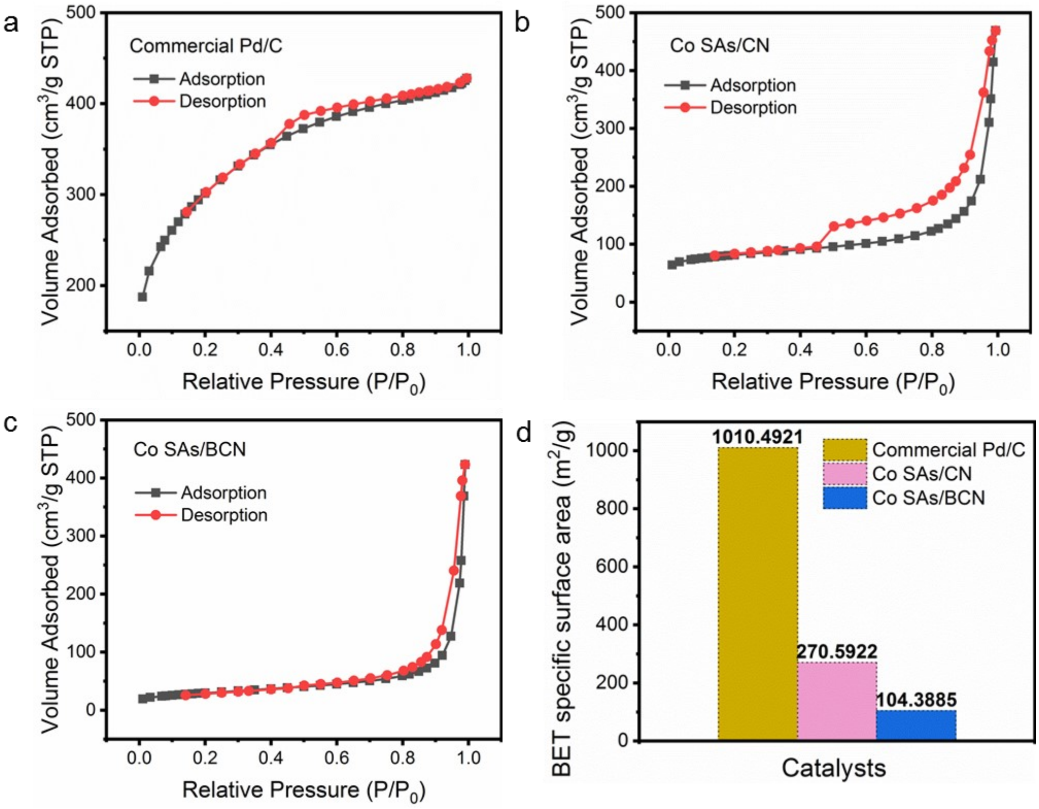

Supplementary Figure 7. N_2 adsorption and desorption isotherm and BET specific surface area. a-c, N_2 adsorption and desorption isotherm for commercial Pd/C, Co SAs/CN and Co SAs/BCN, respectively. **d,** BET specific surface area.

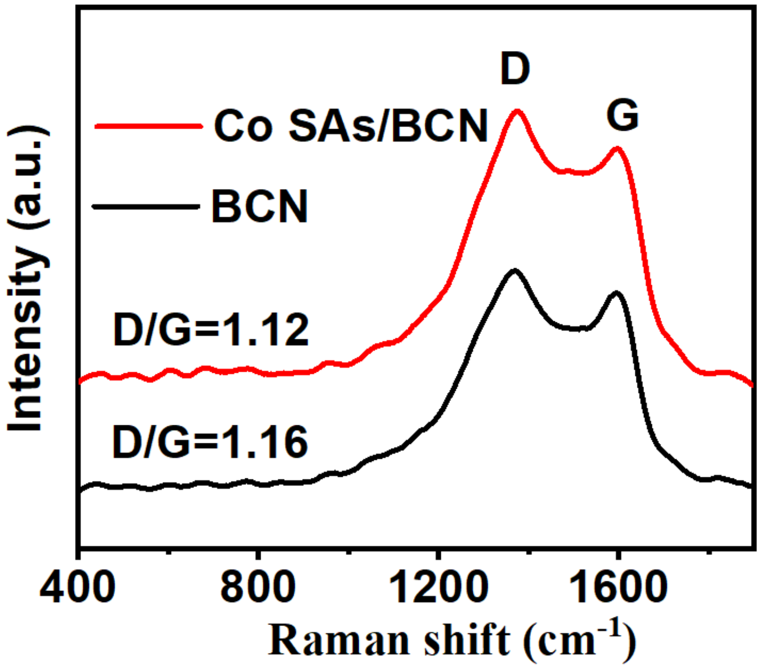

Supplementary Figure 8. Raman spectra of BCN and Co SAs/BCN.

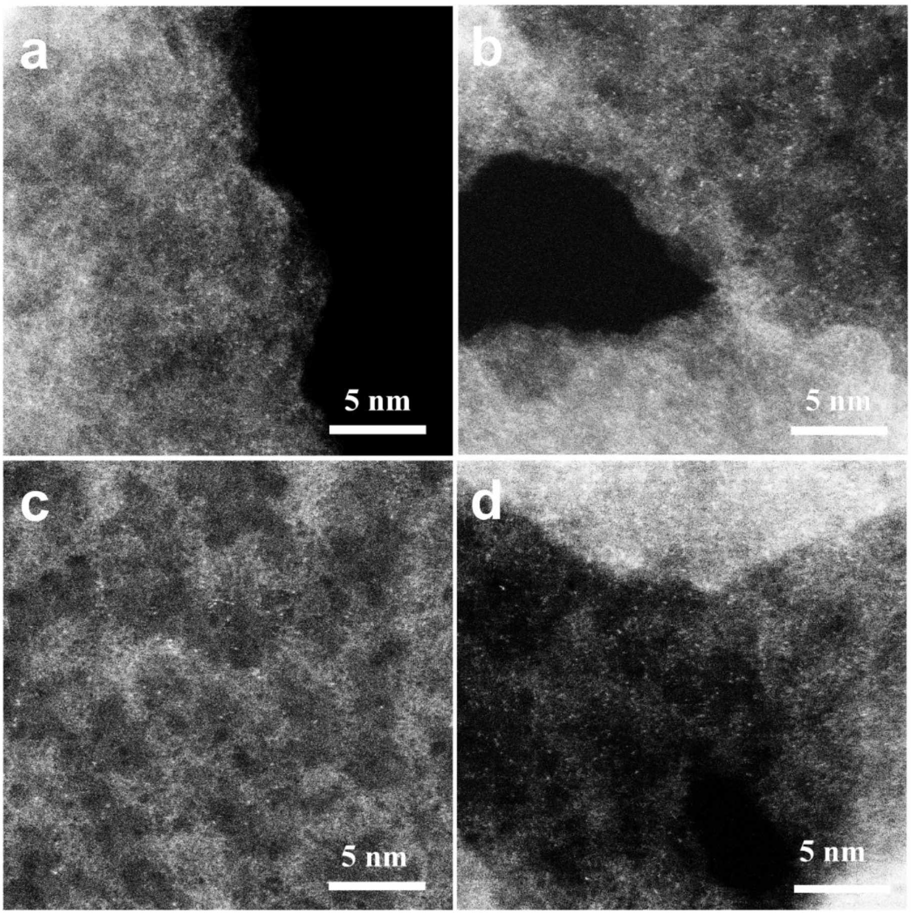

Supplementary Figure 9. Four representative HAADF STEM images of Co SAs/BCN at different areas (a-d).

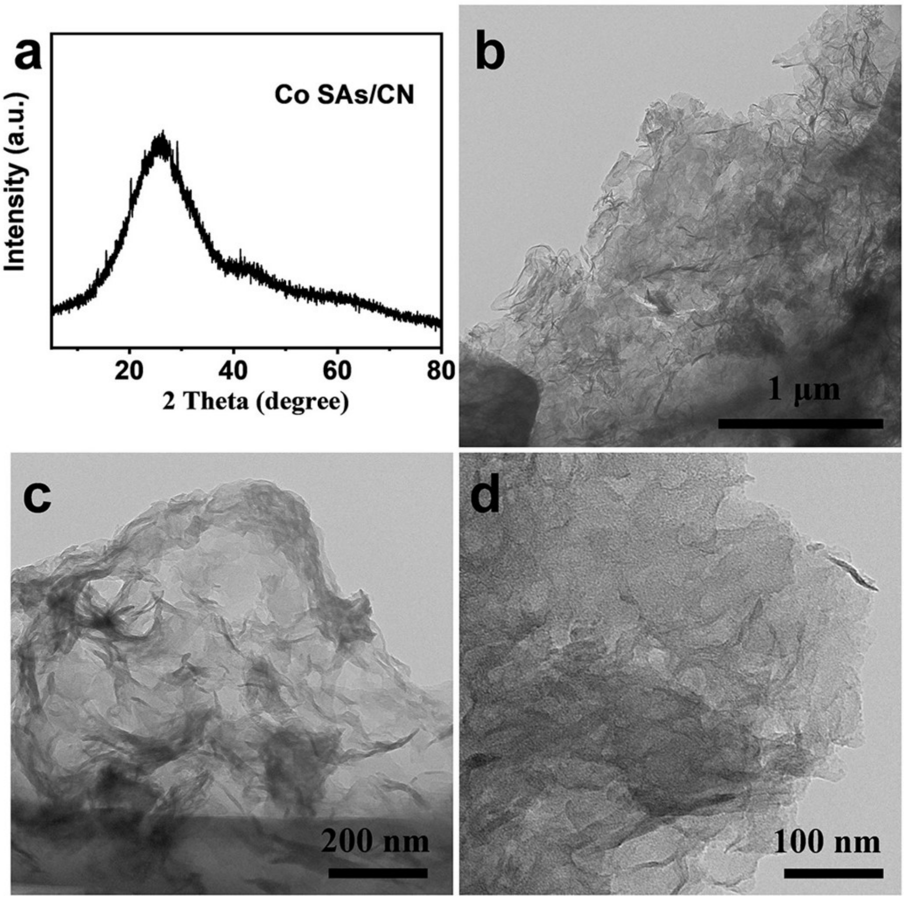

Supplementary Figure 10. Characterizations of Co SAs/CN. a, XRD pattern of Co SAs/CN. **b-d,** Representative TEM images of Co SAs/CN at different magnifications.

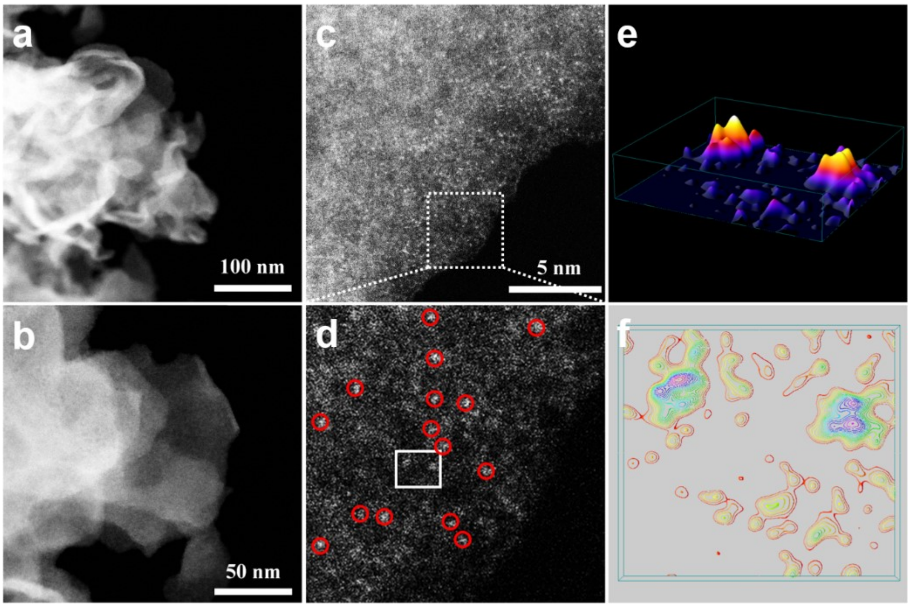

Supplementary Figure 11. HAADF-STEM observation of Co SAs/CN. a, b, HAADF-STEM images of Co SAs/CN at different magnifications. **c, d,** Aberration-corrected HAADF-STEM images of Co SAs/CN. **e,** Atom-overlapping gaussian-function fitting mapping. **f,** 3D isolines of the square from (**d**).

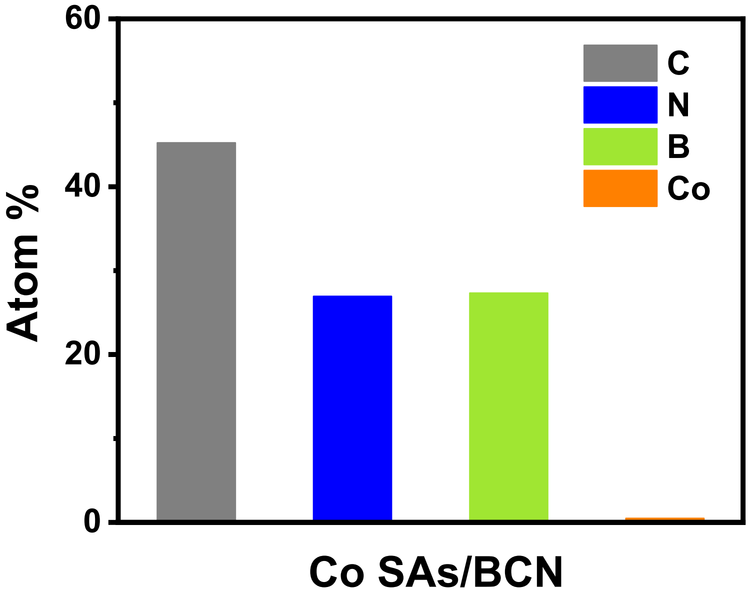

Supplementary Figure 12. Atomic composition of Co SAs/BCN.

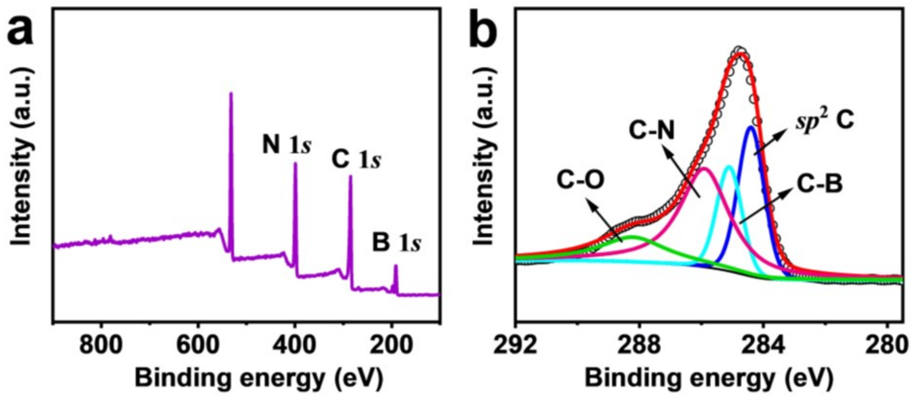

Supplementary Figure 13. XPS spectra of Co SAs/BCN. a, survey scan. **b,** C 1*s* regions of Co SAs/BCN.

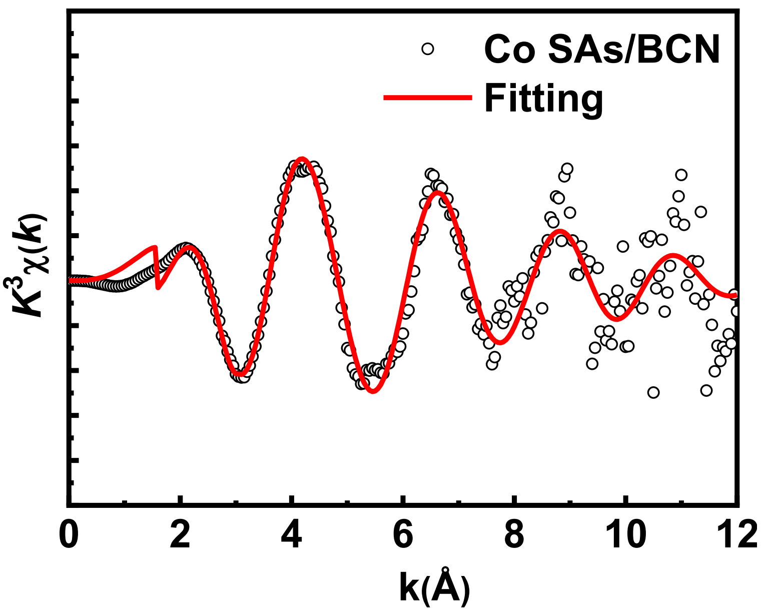

Supplementary Figure 14. The corresponding Co K-edge EXAFS fitting curves of Co SAs/BCN.

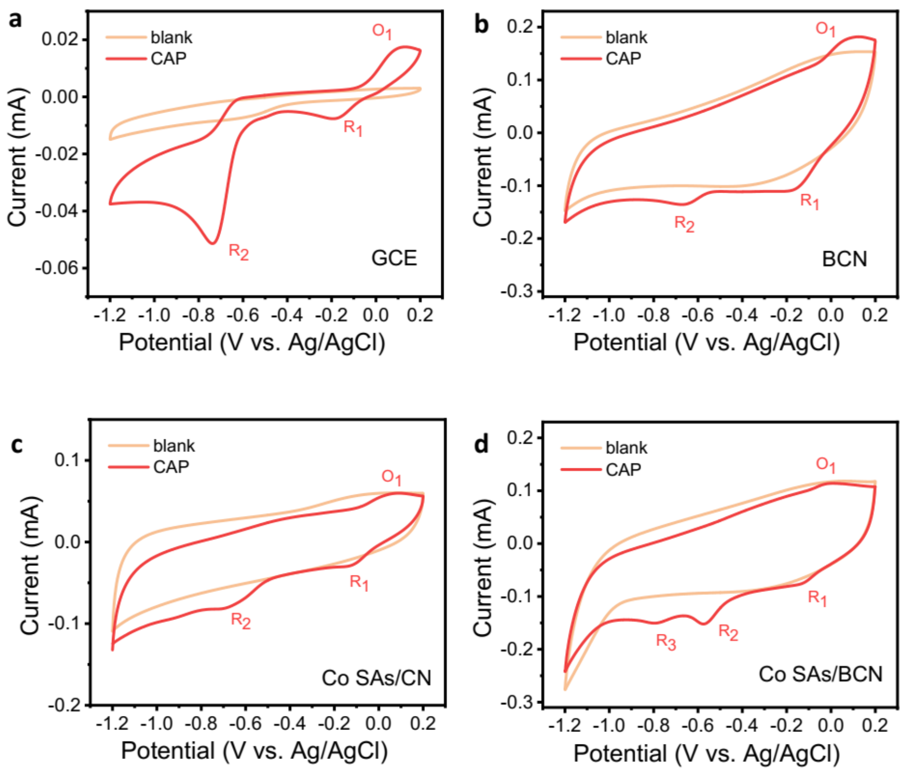

Supplementary Figure 15. Cyclic voltammograms on various cathode electrodes. **a,** GCE, **b,** BCN, **c,** Co SAs/CN, **d,** Co SAs/BCN with/without CAP (scan rate: 100 mV/s).

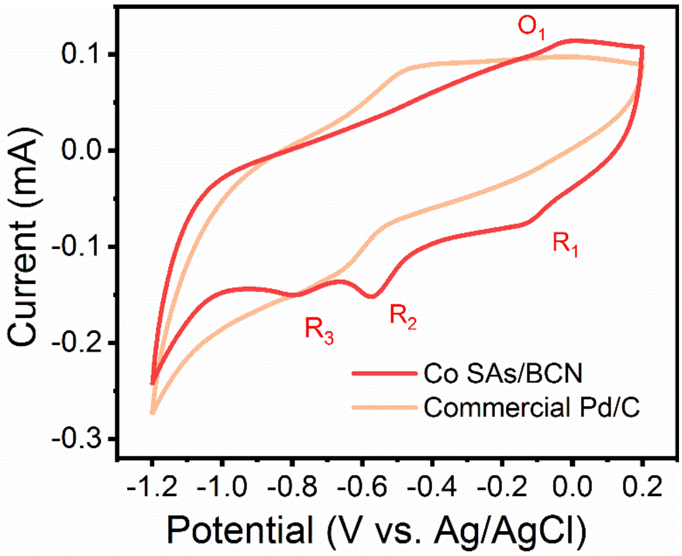

Supplementary Figure 16. Cyclic voltammograms on commercial Pd/C and Co SAs/BCN with CAP (scan rate: 100 mV/s).

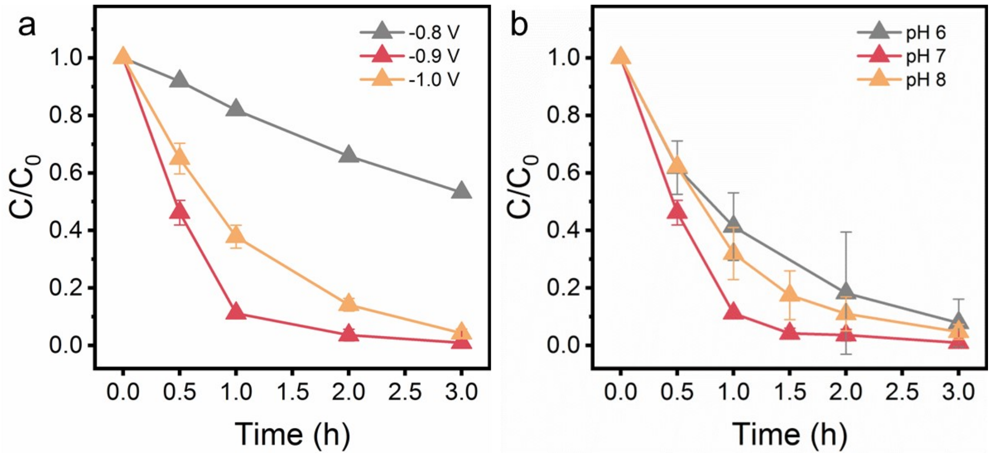

Supplementary Figure 17. Conversion efficiency of CAP over Co SAs/BCN catalysts. a, on various cathode potentials, **b,** at various pHs. The initial concentration of CAP was 50 mg/L. The potential-dependence dechlorination was carried out at pH 7.0. The pH-dependent dechlorination was carried out at -0.9 V.

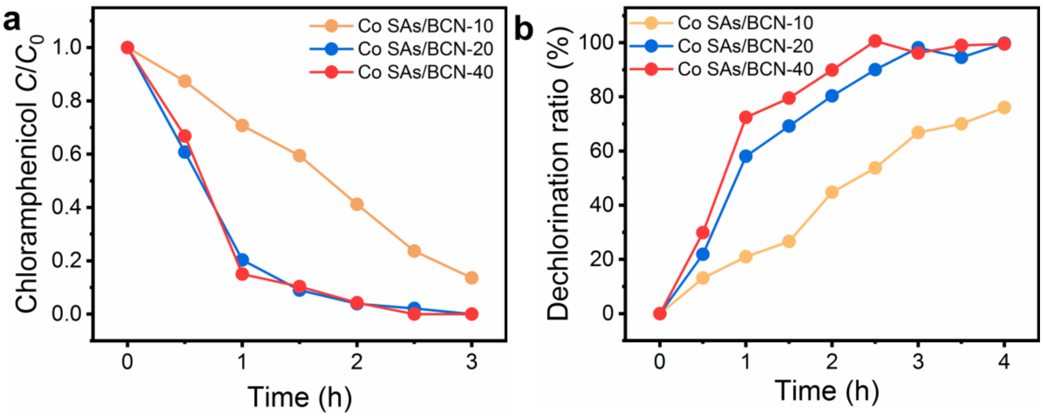

Supplementary Figure 18. Electrocatalytic performance of Co SAs/BCN with different Co doping contents. a, Conversion efficiency, and **b,** dechlorination ratio of CAP on various catalysts.

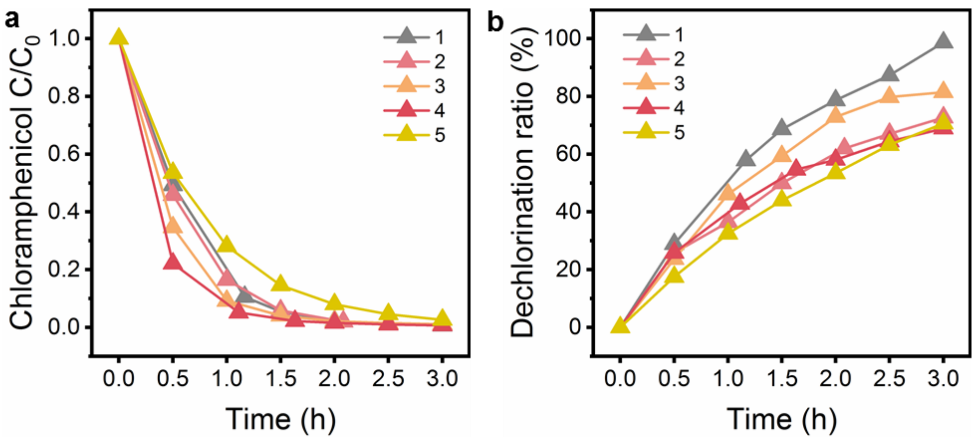

Supplementary Figure 19. Electrocatalytic stability of Co SAs/BCN. a, Conversion efficiency, and **b,** dechlorination ratio of CAP over Co SAs/BCN for five times (15 h) electrolysis at -0.9 V.

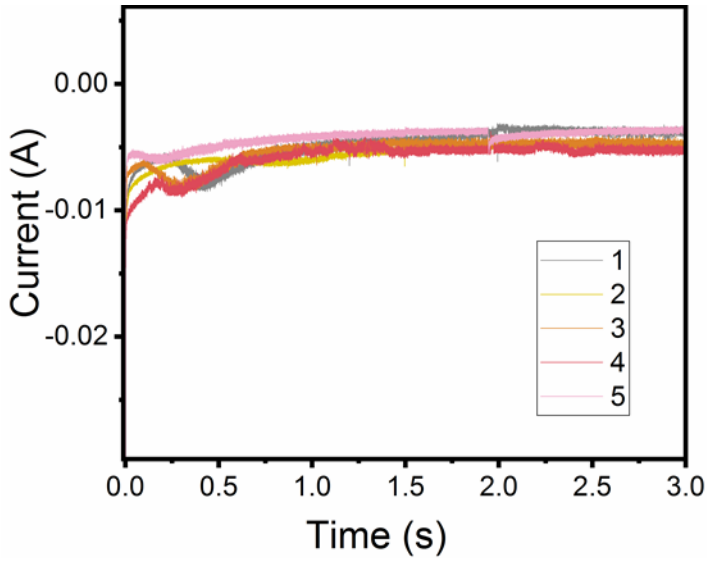

Supplementary Figure 20. The current-time curves of CAP over Co SAs/BCN for five times (15 h) electrolysis at -0.9 V.

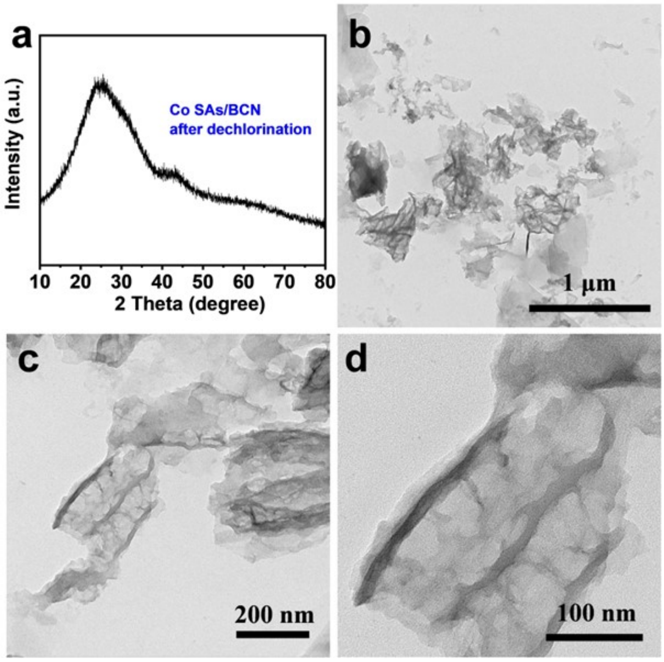

Supplementary Figure 21. Characterizations of Co SAs/BCN after dechlorination. a, XRD and **b-d,** TEM images of Co SAs/BCN after 15 h electrocatalytic dechlorination electrolysis.

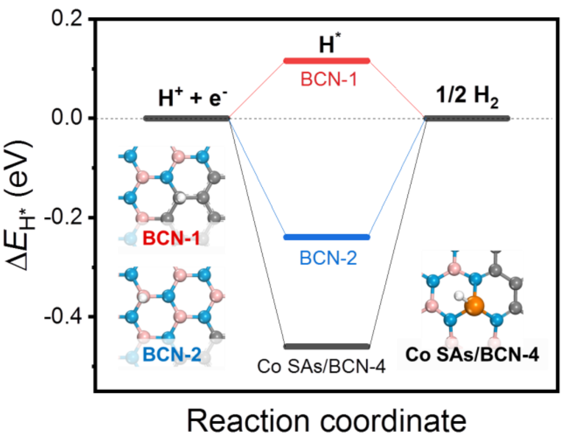

Supplementary Figure 22. Three-state diagram of HER at equilibrium. Red line: H adsorption (H^*) on the C next to N; blue line: H^* on the B; black line: H^* on the Co. Insert: the optimized structures of H* on the three adsorption sites.

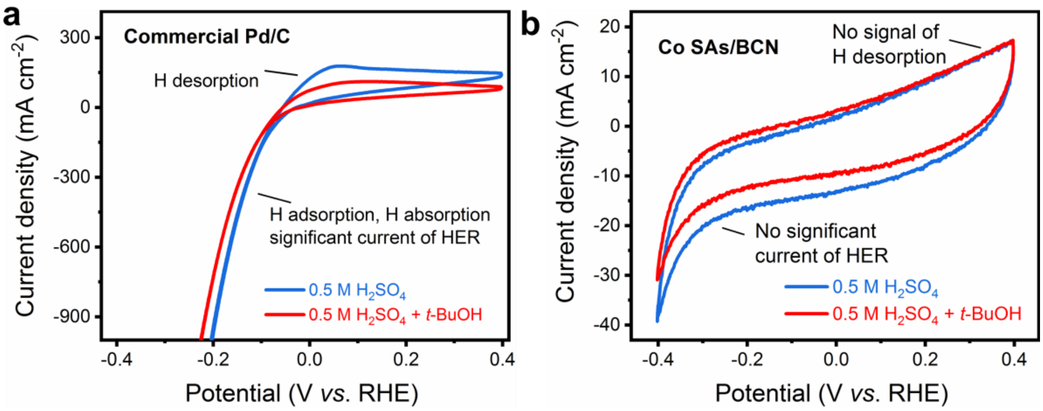

Supplementary Figure 23. Dechlorination mechanism on the Co SAs/BCN electrode and the Pd/C electrode. a, CV curves commercial Pd/C, and **b,** Co SAs/BCN, respectively, in solutions of blank 0.5 M H_2SO_4, or with addition of *t*-BuOH. The dechlorination mechanism on the Co SAs/BCN electrode was independent on H*, which is different from the hydrodechlorination mechanism on the Pd/C electrode.

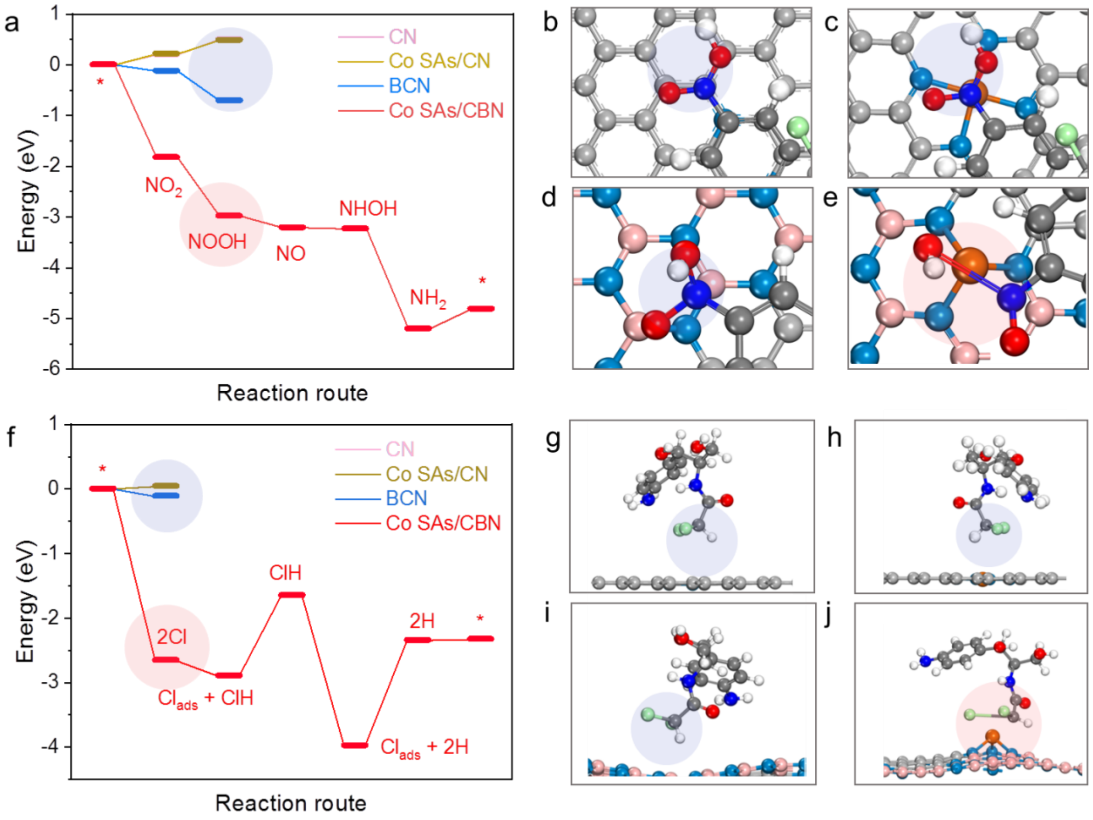

Supplementary Figure 24. Energy diagrams and intermediate structures of CAP conversion on CN, Co SAs/CN, BCN and Co SAs/BCN. a, f, Energy diagrams of $–NO_2$ group reduction and C-Cl bond cleavage of CAP on CN, Co SAs/CN, BCN and Co SAs/BCN, respectively. **b-e,** The adsorption states of the first hydrogenation step during $–NO_2$ group reduction, **g-j,** the first adsorption states of CAP during C-Cl bond reduction after converting $–NO_2$ group to $–NH_2$ group on CN, Co SAs/CN, BCN and Co SAs/BCN, respectively.

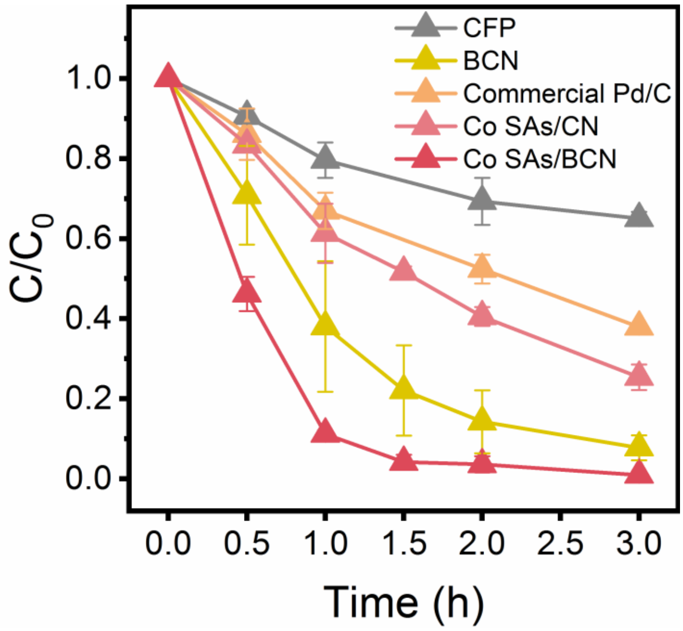

Supplementary Figure 25. Conversion efficiency of CAP on various catalysts.

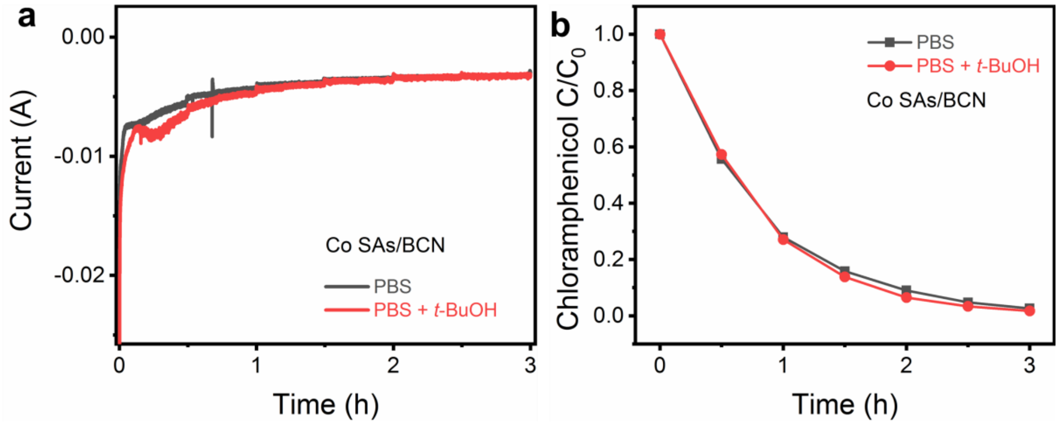

Supplementary Figure 26. Electrocatalytic performance of CAP on the Co SAs/BCN after dosing *tert*-butanol (TBA, *t*-BuOH) in the potentiostatic experiments. **a,** The current-time curves, and **b,** conversion efficiency of CAP over Co SAs/BCN in solutions of blank PBS (pH 7.0), and with adding of *t*-BuOH (10.0 mM).

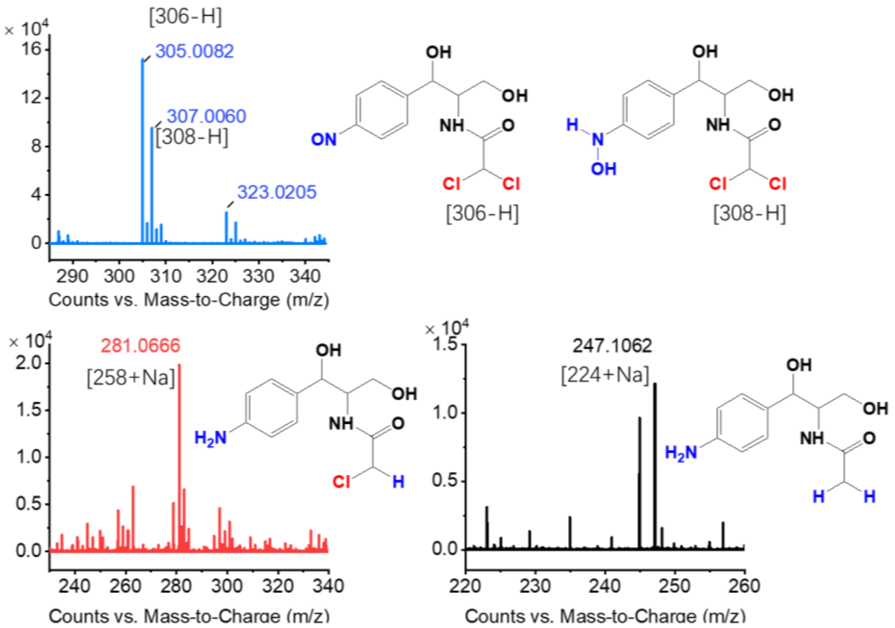

Supplementary Figure 27. The MS identification of CAP reduction products over Co SAs/BCN after 1 h electrolysis ($[CAP]_0$ = 600 ppm).

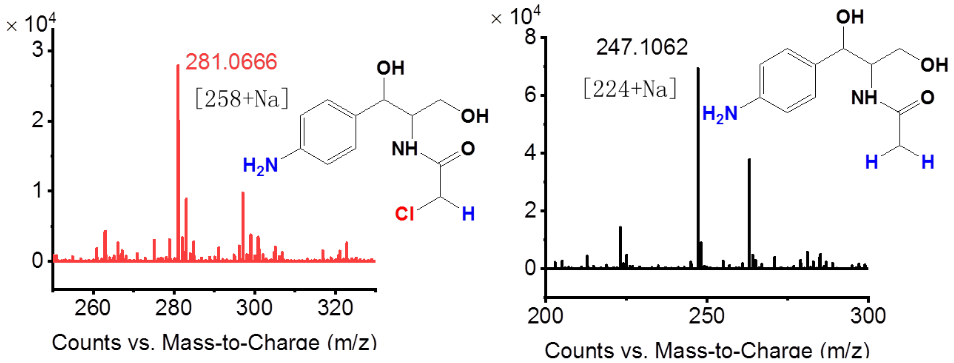

Supplementary Figure 28. The MS identification of CAP reduction products over Co SAs/BCN after 2 h electrolysis ($[CAP]_0$ = 600 ppm).

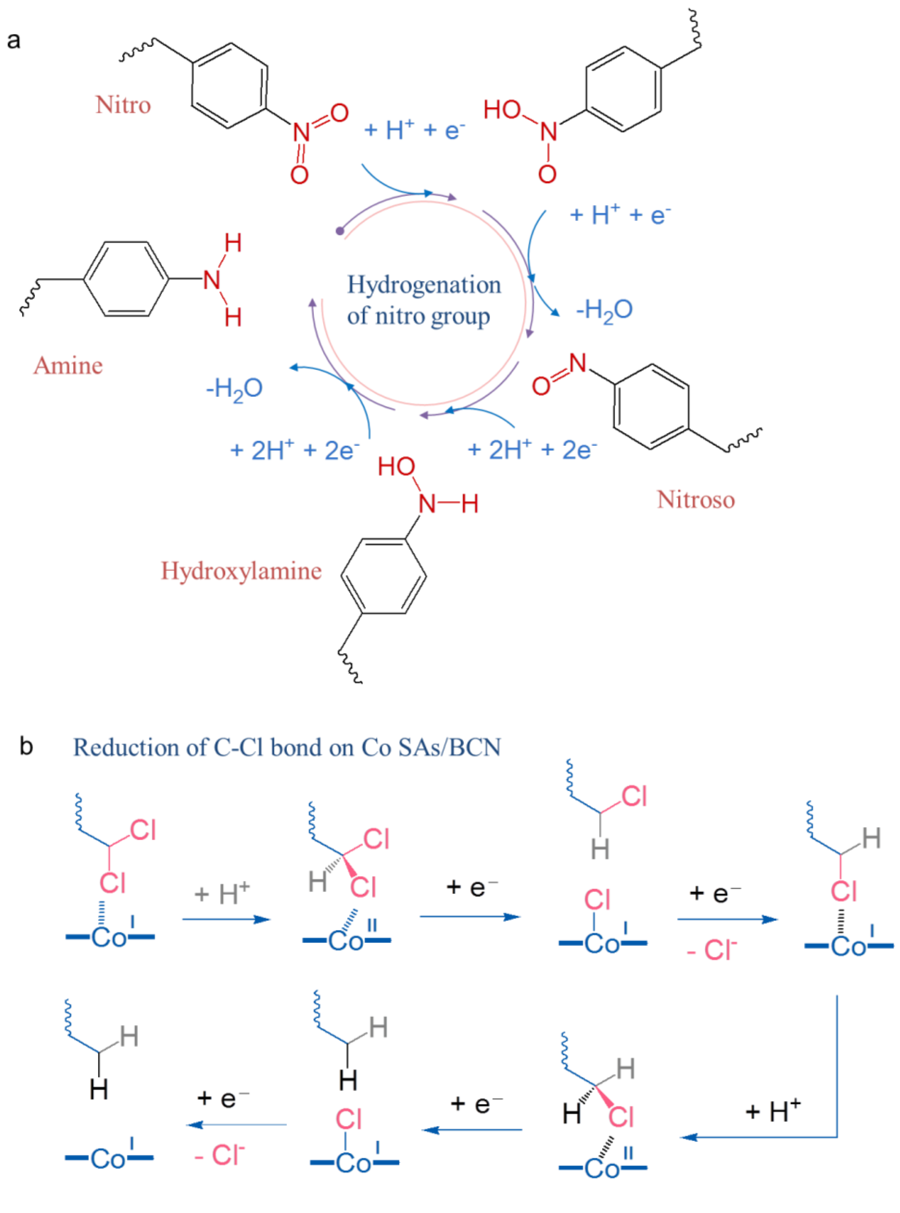

Supplementary Figure 29. Dechlorination mechanisms. a, The typical hydrodechlorination mechanism of -NO_2 group. **b,** The dechlorination mechanism of heterolytic cleavage coupled electron transfer on Co SAs/BCN.

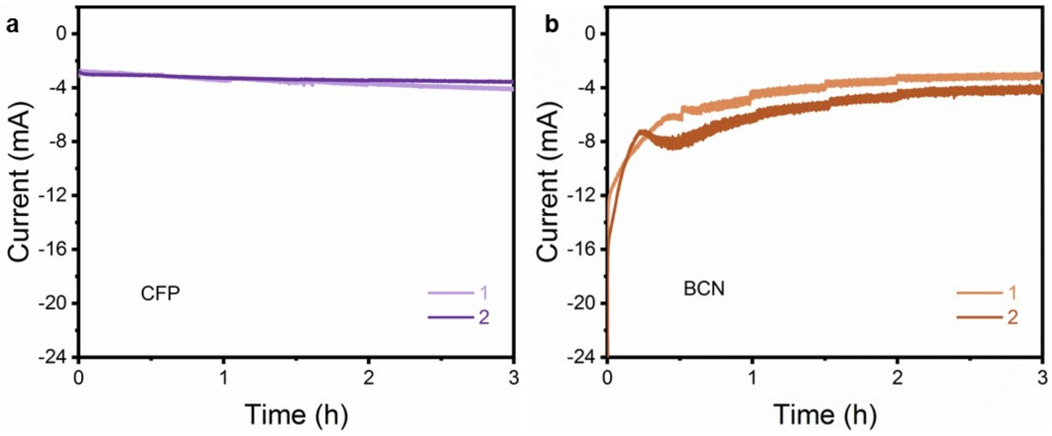

Supplementary Figure 30. The *i-t* curves of control electrodes in CAP solutions. a, carbon fiber paper (CFP), and **b,** BCN (initial concentration of 50 mg/L, pH 7.0, -0.9 V *vs*. Ag/AgCl).

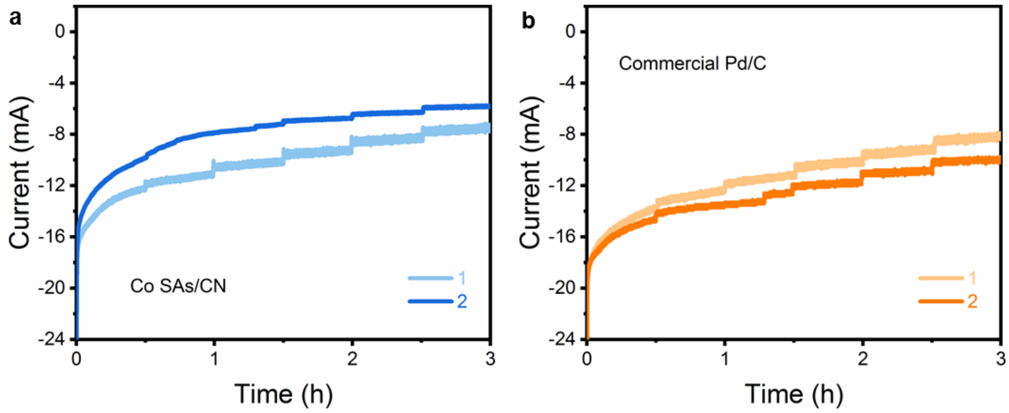

Supplementary Figure 31. The *i-t* curves of different electrodes in CAP solutions. **a,** Co SAs/CN, and **b,** Commercial Pd/C (initial concentration of 50 mg/L, pH 7.0, -0.9 V *vs*. Ag/AgCl).

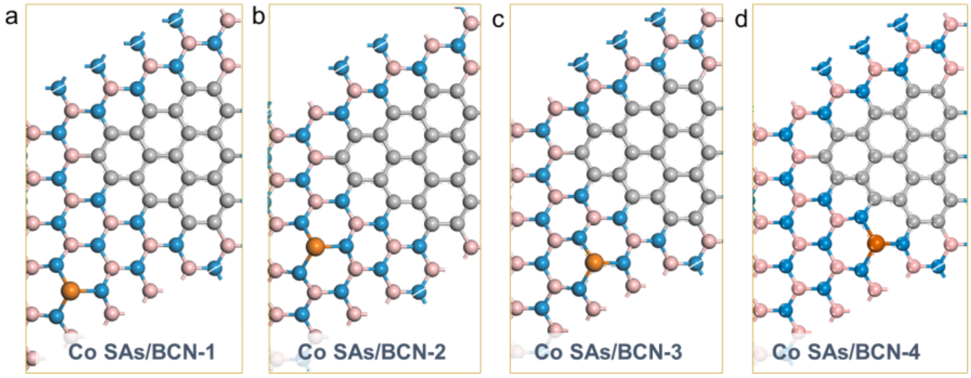

Supplementary Figure 32. BCN support to anchor the Co single atom at possible sites. a, Co SAs/BCN-1, **b,** Co SAs/BCN-2, **c,** Co SAs/BCN-3, **d,** Co SAs/BCN-4. The Co SAs/BCN-4 is the reasonable surface model with the Co active site at the appropriate position.

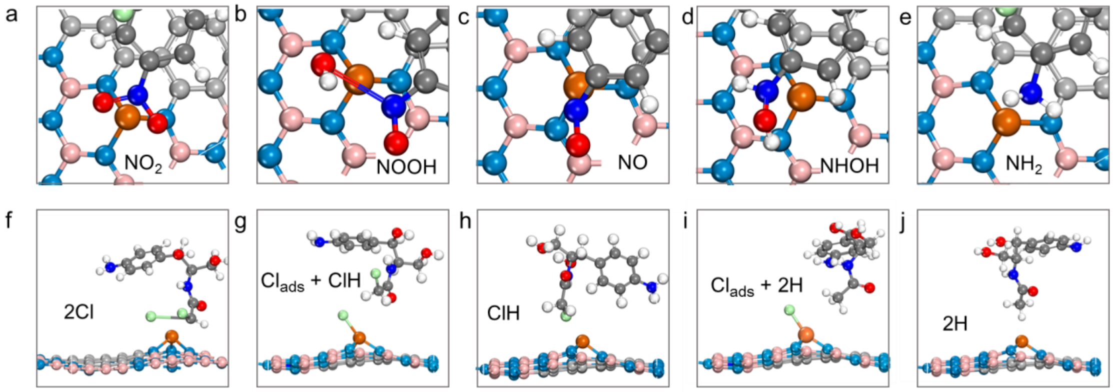

Supplementary Figure 33. Optimized intermediates in the overall reductive dechlorination of CAP Co SAs supported on C-doped boron nitride (Co SAs/BCN). a-e, Reduction of $–NO_2$ group, and **f-j,** dechlorination.

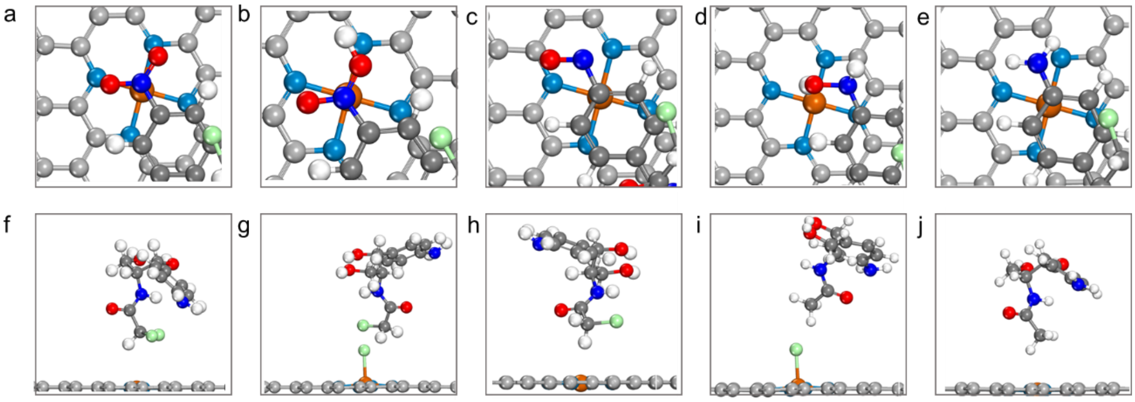

Supplementary Figure 34. Optimized intermediates in the overall reductive dechlorination of CAP on Co SAs supported on N-doped carbon (Co SAs/CN). **a-e,** Reduction of –NO_2 group, and **f-j,** dechlorination

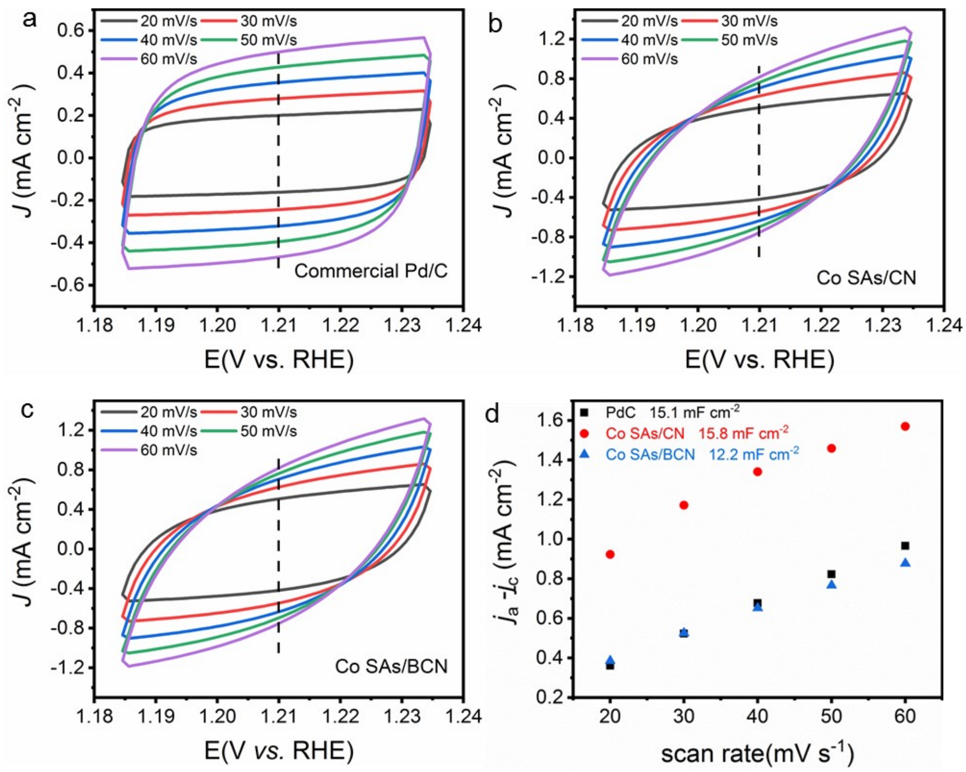

Supplementary Figure 35. Non-Faradic double layer capacitance measurements. **a-c,** CV curves at different scan rates for commercial Pd/C, Co SAs/CN, and Co SAs/BCN, respectively. **d,** Capacitive $\Delta j = j_a - j_c$ as a function of the scan rate of catalysts.

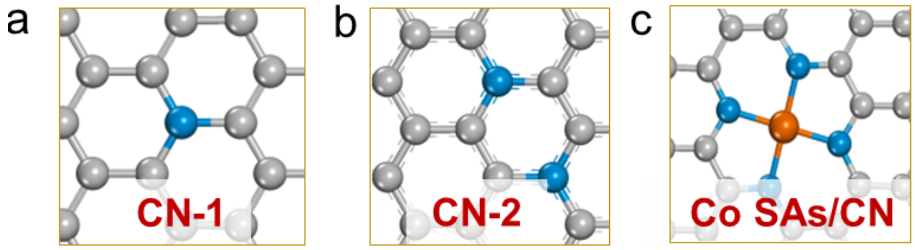

Supplementary Figure 36. Structural characteristics of different models. a-b, N-doped carbon supports, **c,** Co SAs supported on N-doped carbon.

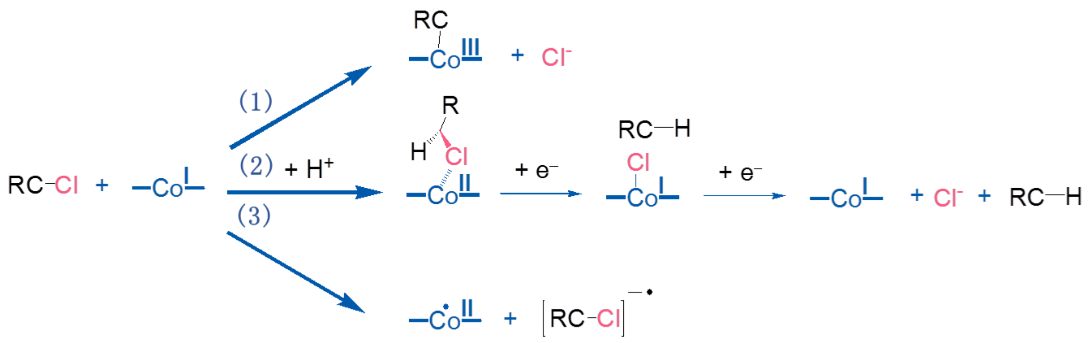

Supplementary Figure 37. Three different reaction mechanisms for dechlorination of dehalogenases.

Supplementary Table 1. EXAFS data fitting results of Co SAs/BCN.

**sample**	**Scattering pair**	**CN**	***R*(Å)**	**σ^2(10^{-3}Å^2)**	**ΔE_0(eV)**	**R factor**
Co SA	Co-N	3.1	2.01	6.2	1.5	0.005

S02 is the amplitude reduction factor; CN is the coordination number; R is interatomic distance (the bond length between central atoms and surrounding coordination atoms); σ^2 is Debye-Waller factor (a measure of thermal and static disorder in absorber-scatterer distances); ΔE_0 is edge-energy shift (the difference between the zero kinetic energy value of the sample and that of the theoretical model). R factor is used to value the goodness of the fitting.

Error bounds that characterize the structural parameters obtained by EXAFS spectroscopy were estimated as N ± 20%; R ± 1%; σ^2 ± 20%; ΔE_0 ± 20%.

Supplementary Table 2. The Comparison of dechlorination performance of heterogeneous catalyst for CAP.

**Catalyst**	**CAP reduction**	**C-Cl removal**	**Method**	**$[CAP]_0$ (mg/L)**	**ratio[a]**	***k*[b] (h^{-1})**	***k*/ratio[c] (h^{-1})**	**Ref.**
**Co SAs/BCN**	100%, 3 h	98%, 3 h	-0.9V *vs.* Ag/AgCl	50	0.28%	1.6963	605.8	Our work
**Co SAs/CN**	75%, 3 h	46%, 3 h	-0.9V *vs.* Ag/AgCl	50	0.28%	0.4600	164.3	Our work
**Commercial Pd/C**	62%, 3 h	50%, 3 h	-0.9V *vs.* Ag/AgCl	50	1.0%	0.3205	32.0	Our work
**CeMo-GO**	99%, 50 min	None Cl^- measured	visible irradiation	20	833%	2.7776* (85%, 40 min)	0.3	ACS Appl. Mater. Interfaces **2017**, 9, 6547-6559
**Nanoscale zero-valent iron NPs**	98%, 6 min	None Cl^- measured	Chemical reduction	97	103.7%	43.0930* (98%, 6 min)	41.6	Chem. Eng. J. **2018**, 334, 508-518
**Biocathode**	87%, 4 h	None Cl^- measured	-0.7 V *vs.* Ag/AgCl	32	/	0.5044* (87%, 4 h)	/	Environ. Sci. Technol. **2013**, 47, 5353−5361
**Graphite fiber brush**	99%, 12 h	75.73 %, 48h (200 mg/L CAP)	-1.25 V *vs.* Ag/AgCl	50	/	0.6746* (98%, 6 h)	/	Water Res. **2015**, 72, 281-292

[a]Metal / CAP mass ratio: utilization efficiency of metals in the catalyst. [b]Rate constant (*k*) of CAP reduction. The conversion efficiency of CAP and the corresponding reaction time are listed in parentheses. [c]Rate constant (*k*) contributed by unit mass of metal. *The values were fitted by the data extracted from the references using GetData Graph Digitizer software.

Supplementary Note 1

The important factor of surface area or electrochemically active area was measured to evaluate the electrochemical catalytic activity. Non-Faradic double layer capacitance (C_{dl}) was measured to compare electrochemical surface area (ECSA) by cyclic voltammetry (CV) at the non-Faradic region (Supplementary Fig. 35). The results indicate that two control samples of the Co SAs/CN and commercial Pd/C with carbon-based supports exhibited similar C_{dl}, but the Co SAs/BCN had a smaller one. Generally, a smaller C_{dl} indicates a smaller ECSA. We also measured the Brunauer-Emmett-Teller (BET) specific surface area (SSA) of the three samples (Supplementary Fig. 7). The results show that the Co SAs/CN (270.5922 m^2/g) and the commercial Pd/C (1,010.4921 m^2/g) had a larger SSA than our Co SAs/BCN (104.3885 m^2/g). Therefore, the current density normalized by SSA was also the highest among the tested materials, and the Co SAs/BCN had obvious advantages in electrocatalytic activity.

However, the ECSA measurements by C_{dl} might generate confusion for some new types of electrocatalyst. For example, SAC has a low metal loading, thus the double layer capacitance contributed by support is far more than that by metal atoms. But the catalytic capability is mainly ascribed to the highly dispersed metal atoms, instead of support. Therefore, in this case, ECSA might be incapable to evaluate the electrocatalytic activity accurately. The ECSA measurements provide information about the number of electrochemically active sites on the electrodes, but not all electrochemically active sites are catalytically active.[1] If the catalyst is in the form of particles, especially in the form of nanoparticles, the roughness factor (estimated by normalizing the double layer capacitance of the electrode with the double layer capacitance of a flat surface, ECSA = C_{dl}/C_{dlRef}) can be very high.[2] Therefore, the current density normalized by ECSA ($j_{ECSA} = j$/ECSA) is useful to evaluate the electrocatalytic activity for nanoparticle materials, but is not suitable for single-atom catalysts.

Supplementary Note 2

For dehalogenases, three different reaction mechanisms for dechlorination have been proposed,[3] including: 1) nucleophilic substitution: the formation of a cobalt–carbon bond after alkylation of the cobalt by a nucleophilic attack of Co on the carbon backbone of the organohalide; 2) heterolytic cleavage coupled electron transfer: the formation of a cobalt–halogen bond after direct Co attack on the halogen substituent; and 3) long-range electron transfer: a long-range electron transfer from Co leading to substrate radical formation followed by the formation of a carbanion after elimination of the halogen substituent (Supplementary Figure 37). As revealed by the structural analysis of dehalogenases, an alkylation of the cobalt during substrate conversion is unlikely due to the spatial restraints caused by the amino acid arrangement at the active site. In this work, the reasonable dechlorination mechanism of the Co SAs/BCN is the heterolytic cleavage coupled electron transfer. The reasons are given as follows:

To verify the different dechlorination mechanisms on the Co SAs/BCN and Pd/C electrode, the CV experiments were carried out in 0.5 M H_2SO_4. The results show that the CV with the Pd/C electrode exhibited the obvious polarization current during scanning in the negative direction (< -0.1 V *vs*. RHE), contributed by H* adsorption, absorption and subsequent H_2 evolution reaction (HER). Correspondingly, an obvious current peak of H* desorption was presented in the positive direction (> 0 V *vs*. RHE) (Supplementary Fig. 23a). This observation agrees well with the results in the literature.[2] However, in the same potential range, there was no significant current of HER and no signal of H* desorption on the CV with the Co SAs/BCN electrode (Supplementary Fig. 23b). This result indicates that the Co SAs/BCN was not beneficial to generate H*. Nevertheless, the Co SAs/BCN electrode showed a higher dechlorination capability than the Pd/C electrode. Thus, the dechlorination on the Co SAs/BCN electrode was independent on H*, which is different from the hydrodechlorination mechanism on the Pd/C electrode. This is also verified by adding *tert*-butanol (TBA), an effective H* scavenger, into the solution.[4] After the dose of

approximately 100.0 mM TBA, an immediate quenching of the H* oxidation peak was observed on the Pd/C electrode. The suppressed H* generation markedly inhibited the dechlorination performance on the Pd/C electrode (Fig. 4g), but had no effect on the dechlorination capability of the Co SAs/BCN (Fig. 4h). In addition, the electrocatalytic performance of CAP on the Co SAs/BCN was also not affected after dosing TBA in the potentiostatic experiments (Supplementary Fig. 26). Therefore, the Co SAs/BCN inspired by the dehalogenase had a different dechlorination mechanism from the traditional electrocatalysts.

Supplementary Note 3

The CAP degradation mechanism by the Co SAs/BCN and Co SAs/CN is heterolytic cleavage, but that by the Pd/C catalyst is hydrodechlorination mechanism with the first step of H* generation reported in literature.[5-7] However, the different mechanisms for CAP reduction include the same steps: NO_2 reduction and dechlorination. Thus, the degradation products by the Co SAs/BCN, Co SAs/CN and Pd/C should be consistent with the previous report.[8] Simultaneously, the corresponding products of CAP by the Co SAs/BCN were also determined by the HPLC/MS (Supplementary Fig. 27-28). The results show that four main products were identified in the initial 1 h, i.e., nitroso products (NO−R−Cl_2 with $[M-H]^-$ at *m*/*z* 305), hydroxylamine products (NHOH−R−Cl_2 with $[M-H]^-$ at *m*/*z* 307) and aromatic amine products (NH_2−R−ClH with $[M+Na]^+$ at *m*/*z* 281, NH_2−R−H_2 with $[M+Na]^+$ at *m*/*z* 247). After extending a reaction time to 2 h, only the aromatic amine products with one Cl atom (NH_2−R−ClH) and without any Cl atoms (NH_2−R−H_2) were identified. Although the dechlorination mechanisms are different, all the intermediates generated on the Co SAs/BCN were consistent with the results from Pd/C through the same steps of NO_2 reduction and dechlorination (Supplementary Fig. 29).

Supplementary Note 4

Substituted terpyridine was first synthesized by a modified procedure of the

literature.[9] 2-acetylpyridine (37.0 mmol, 4.47 g) and p-Anisaldehyde (18.5 mmol, 2.24 g) benzaldehyde were dissolved in ethanol (40 mL) by stirring in a 100 mL round bottomed flask, followed by addition of (37.0 mmol, 2.08 g) potassium hydroxide, and (6.43 mL, 28 wt.%) of ammonium hydroxide. The mixture was allowed to reflux for 4 h, resulting in the formation of a yellow precipitate. The yellow solid was collected by vacuum filtration and rinsed with cold methanol.

Supplementary methods

DFT calculations. All calculations were carried out with DFT calculations using the CASTEP code.[10] All-electron calculations were employed using the generalized gradient approximation (GGA) and the Perdew, Bueke, and Ernzerhof (PBE) functional.[11] During the geometry optimization, the minimum energies for all structures were obtained until energy became less than 1×10^{-5} eV/atom. The atomic position was fully relaxed until the maximum force and the maximum displacement for each atom was less than 0.03 eV/Å and within 1×10^{-3} Å, respectively. Noncovalent forces, such as hydrogen bonding and van der Waals (vdW) interactions, were described using the empirical correction in the TS scheme.[12] During the electronic minimization, the ultrasoft pseudopotential for each element was used, which is fairly efficient than the norm-conserving pseudopotentials.[13] The plane-wave cutoff energy was 450 eV, and variable occupancy calculations were conducted using density mixing method. Electron smearing of 0.1 eV was used according to the Gaussian scheme. The Self-consistent field (SCF) tolerance was set to 5×10^{-6} eV/atom for the geometrical and electronic optimization.

To model the Co SAs/BCN, we first built a periodic 6×6 supercell containing 36 boron and 36 nitrogen atoms with a vacuum of 18 Å in the z-direction. The subsititutional carbon defects was in a triangular shape by replacing BN with 22 carbon atoms, according to previous synthesis and theoretical studies of carbon doped BN sheet.[14,15] Newly formed C-N and C-B bonds at the boundary linked the BN region and C region. Then, one B atom was removed to create a B monovacancy to provide an anchoring site for a single Co atom. The distance of B-vacancy to the boundary gave rise to various chemical environment of B-vacancy and the captured Co atom. As shown in Supplementary Fig. 32, four kinds of Co SAs/BCN sites were proposed and labeled as CoBCN-1 to 4 to study the catalytic activity of CAP reductive dechlorination. The *k*-point sampling of the Brillioun zone was done using a $2 \times 2 \times 1$ *k*-points grid for electronic structure computations. The Mulliken charge analysis was performed to compute the charge transfer.

Energy profile of the reductive dechlorination process were calculated based on a computational standard hydrogen electrode (SHE) model,[16] similar to the methods used in the literature.[17] Throughout this work we reference the potential relative to the CHE. The energy of each step involving electron transfer is given by

$$\Delta E = \Delta E_{DFT} - eU \qquad (1)$$

where ΔE_{DFT} is the change of total energy from DFT calculations for the intermediate species adsorbed on the BCN or Co SAs/BCN; U is the electrode potential; e is the transferred electron in the reaction. Total energy is useful to predict the trends in reductive dechlorination activity of large catalytic systems, as reported in the literatures.[18] The zero point energy and entropy corrections are much smaller than the total energy with less contribution to the trends of free energy change. The adsorption energy of CAP on the surface (* + CAP → CAP*) was defined by $E_{ads} = E_{CAP*} - (E_{*} + E_{CAP})$, and the adsorption energy of proton ($* + H^+ + e^- \rightarrow H*$) was calculated by $E_{ads} = E_{H*} - (E_{*} + E_{H+} + E_{e-})$, where E_{CAP*} and E_{H*} is the total energy of the surface with CAP and H, respectively; E_{*} is the total energy of the surface, and E_{CAP} is the total energy of the isolated CAP; at zero potential and pH = 0, $E_{H+} + E_{e-}$ is estimated from the half enery of H_2 gas, due to the equlibrium of $H^+ + e^- \rightarrow \frac{1}{2}H_2$. Thus, the more negative the adsorption energy, the stronger the adsorption on the surface. For the desorption of chloride ion, $Cl* + e^- \rightarrow * + Cl^-$, the reaction energy change was calculated by $\Delta E = E_{*} - E_{Cl*} + (E_{Cl-} - E_{e-})$,[19,20] where E_{Cl*} is the total energy of the surface with Cl, and then, according to the reaction of $Cl^- \rightarrow \frac{1}{2}Cl_2 + e^-$, $E_{Cl-} - E_{e-}$ is estimated from $\frac{1}{2}E_{Cl2} - e(U^{\Theta} - U) - kT\ln[Cl^-]$, where U^{Θ} is the standard potential of the reversible chlorine electrode, $U^{\Theta} = 1.36$ V, $[Cl^-]$ is the concentration of chloride ions, k is the Boltzmann constant.

Supplementary References

1. Voiry, D. *et al.* Best practices for reporting electrocatalytic performance of nanomaterials. *ACS Nano* **12**, 9635-9638 (2018).
2. Sun, S., Li, H. & Xu, Z. J. Impact of surface area in evaluation of catalyst activity. *Joule* **2**, 1024-1027 (2018).
3. Kunze, C. *et al.* Cobamide-mediated enzymatic reductive dehalogenation via long-range electron transfer. *Nat. Commun.* **8**, 15858 (2017).
4. Grdeń, M., Łukaszewski, M., Jerkiewicz, G. & Czerwiński, A. Electrochemical behaviour of palladium electrode: Oxidation, electrodissolution and ionic adsorption. *Electrochim. Acta* **53**, 7583-7598 (2008).
5. Liu, R. *et al.* Defect sites in ultrathin Pd nanowires facilitate the highly efficient electrochemical hydrodechlorination of pollutants by H^*_{ads}. *Environ. Sci. Technol.* **52**, 9992-10002 (2018).
6. Mao, R. *et al.* Dechlorination of trichloroacetic acid using a noble metal-free graphene–Cu foam electrode via direct cathodic reduction and atomic H*. *Environ. Sci. Technol.* **50**, 3829-3837 (2016).
7. Durst, J., Siebel, A., Simon, C., Hasché, F., Herranz, J. & Gasteiger, H. A. New insights into the electrochemical hydrogen oxidation and evolution reaction mechanism. *Energy Environ. Sci.* **7**, 2255-2260 (2014).
8. Kong, D. *et al.* Cathodic degradation of antibiotics: Characterization and pathway analysis. *Water Res.* **72**, 281-292 (2015).
9. Stublla, A. & Potvin, P. G. Ruthenium(II) complexes of carboxylated terpyridines and dipyrazinylpyridines. *Eur. J. Inorg. Chem.* **2010**, 3040-3050 (2010).
10. Clark, S. J. *et al.* First principles methods using CASTEP. *Z. Kristallogr.* **220**, 567-570 (2005).
11. Perdew, J. P., Burke, K. & Ernzerhof, M. Generalized gradient approximation made simple. *Phys. Rev. Lett.* **77**, 3865-3868 (1996).
12. Tkatchenko, A. & Scheffler, M. Accurate molecular Van der Waals interactions from ground-state electron density and free-atom reference data. *Phys. Rev. Lett.* **102** (2009).
13. Vanderbilt, D. Soft self-consistent pseudopotentials in a generalized eigenvalue formalism. *Phys. Rev. B* **41**, 7892-7895 (1990).
14. Huang, C. J. *et al.* Carbon-doped BN nanosheets for metal-free photoredox catalysis. *Nat. Commun.* **6** (2015).
15. Berseneva, N., Gulans, A., Krasheninnikov, A. V. & Nieminen, R. M. Electronic structure of boron nitride sheets doped with carbon from first-principles calculations. *Phys. Rev. B* **87** (2013).
16. Man, I. C. *et al.* Universality in oxygen evolution electrocatalysis on oxide surfaces. *Chemcatchem* **3**, 1159-1165 (2011).
17. Exner, K. S., Anton, J., Jacob, T. & Over, H. Controlling selectivity in the chlorine evolution reaction over RuO_2-based catalysts. *Angew Chem., Int. Edit.* **53**, 11032-11035 (2014).

18. Kattel, S., Atanassov, P. & Kiefer, B. Stability, electronic and magnetic properties of in-plane defects in graphene: a first-principles study. *J. Phys. Chem. C* **116**, 8161-8166 (2012).
19. Vittadini, A., Selloni, A., Rotzinger, F. P. & Gratzel, M. Formic acid adsorption on dry and hydrated TiO_2 anatase (101) surfaces by DFT calculations. *J. Phys. Chem. B* **104**, 1300-1306 (2000).
20. Hansen, H. A. *et al.* Electrochemical chlorine evolution at rutile oxide (110) surfaces. *Phys. Chem. Chem. Phys.* **12**, 283-290 (2010).
