# Supplementary material for: Integrating single-cobalt-site and electric field of boron nitride in dechlorination electrocatalysts by bioinspired design
Source: Nat Commun. 2021 Jan 12;12:303. doi: 10.1038/s41467-020-20619-w (PMC7803959; doi:10.1038/s41467-020-20619-w)
Supplement: Supplementary file 2 — Description of Additional Supplementary Files [file 41467_2020_20619_MOESM2_ESM.pdf]

Description of Additional Supplementary Files

File Name: Supplementary Movie 1

Description: X-ray tomography 3D reconstruction of Co SAs/BCN
